# Supplementary material for: Pre-treatment untargeted cerebrospinal fluid metabolomic profiling in tuberculous meningitis uncovers pathways associated with mortality
Source: Med. Author manuscript; Available in PMC 2026 Feb 19. (PMC7618751; doi:10.1016/j.medj.2025.100703)
Supplement: Supplementary Materials [file EMS212236-supplement-Supplementary_Materials.zip › 1-s2.0-S2666634025001308-mmc1.pdf]

Med, Volume *6*

Supplemental information

Pre-treatment untargeted cerebrospinal fluid metabolomic profiling in tuberculous meningitis uncovers pathways associated with mortality

Thanh Hoang Nhat Le, Kirsten C.J. van Abeelen, Edwin Ardiansyah, Julian Avila-Pacheco, Sofiati Dian, Gesa Carstens, Lara Schramke, Hoang Thanh Hai, Tran Binh Minh Nguyen, Thai Minh Triet, Amy Deik, Jesse Krejci, Jeff Pruyne, Lucas Dailey, Bachti Alisjahbana, Mihai G. Netea, Riwanti Estiasari, Trinh Thi Bich Tram, Joseph Donovan, Dorothee Heemskerk, Thi Hong Chau Tran, Nguyen Duc Bang, Ahmad Rizal Ganiem, Raph L. Hamers, Rovina Ruslami, Darma Imran, Kartika Maharani, Vinod Kumar, Reinout van Crevel, Guy Thwaites, Clary B. Clish, Nguyen Thuy Thuong Thuong, and Arjan van Laarhoven

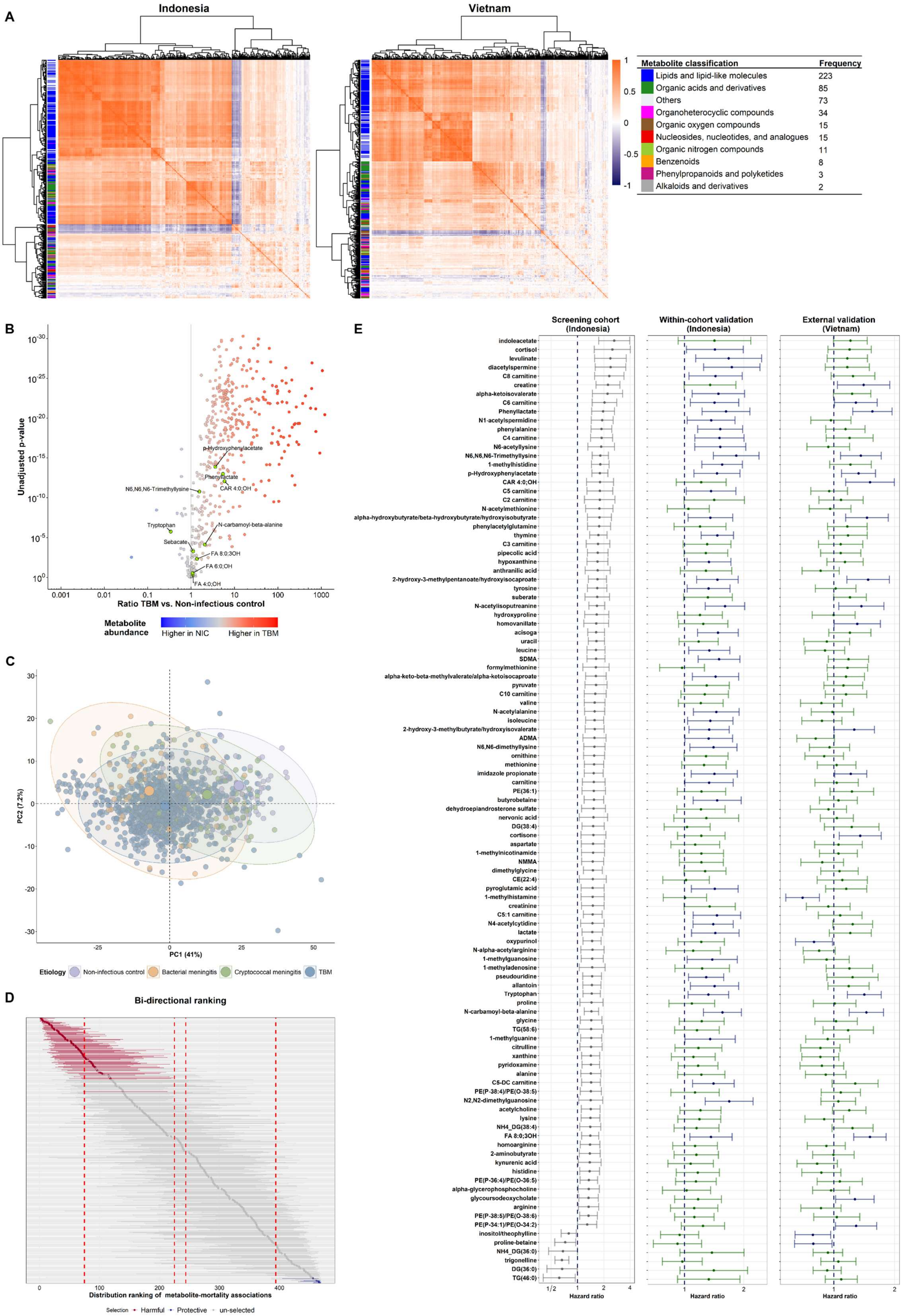
A
Indonesia
Vietnam
1
0.5
0
-0.5
-1
Metabolite classification
Frequency
Lipids and lipid-like molecules 223
Organic acids and derivatives 85
Others 73
Organoheterocyclic compounds 34
Organic oxygen compounds 15
Nucleosides, nucleotides, and analogues 15
Organic nitrogen compounds 11
Benzenoids 8
Phenylpropanoids and polyketides 3
Alkaloids and derivatives 2
B
Unadjusted p-value
p-Hydroxyphenylacetate
Phenyllactate
CAR 4:0;OH
N6,N6,N6-Trimethyllysine
Tryptophan
N-carbamoyl-beta-alanine
Sebacate
FA 8:0;3OH
FA 6:0;OH
FA 4:0;OH
Ratio TBM vs. Non-infectious control
Metabolite abundance
Higher in NIC
Higher in TBM
C
PC2 (7.2%)
PC1 (41%)
Etiology
Non-infectious control
Bacterial meningitis
Cryptococcal meningitis
TBM
D
Bi-directional ranking
Distribution ranking of metabolite-mortality associations
Selection
Harmful
Protective
un-selected
E
Screening cohort (Indonesia)
Within-cohort validation (Indonesia)
External validation (Vietnam)
indoleacetate
cortisol
levulinate
diacetylspermine
C8 carnitine
creatine
alpha-ketoisovalerate
C6 carnitine
Phenyllactate
N1-acetylspermidine
phenylalanine
C4 carnitine
N6-acetyllysine
N6,N6,N6-Trimethyllysine
1-methylhistidine
p-Hydroxyphenylacetate
CAR 4:0;OH
C5 carnitine
C2 carnitine
N-acetylmethionine
alpha-hydroxybutyrate/beta-hydroxybutyrate/hydroxyisobutyrate
phenylacetylglutamine
thymine
C3 carnitine
pipecolic acid
hypoxanthine
anthranilic acid
2-hydroxy-3-methylpentanoate/hydroxyisocaproate
tyrosine
suberate
N-acetylisoputreanine
hydroxyproline
homovanillate
acisoga
uracil
leucine
SDMA
formylmethionine
alpha-keto-beta-methylvalerate/alpha-ketoisocaproate
pyruvate
C10 carnitine
valine
N-acetylalanine
isoleucine
2-hydroxy-3-methylbutyrate/hydroxyisovalerate
ADMA
N6,N6-dimethyllysine
ornithine
methionine
imidazole propionate
carnitine
PE(36:1)
butyrobetaine
dehydroepiandrosterone sulfate
nervonic acid
DG(38:4)
cortisone
aspartate
1-methylnicotinamide
NMMA
dimethylglycine
CE(22:4)
pyroglutamic acid
1-methylhistamine
creatinine
C5:1 carnitine
N4-acetylcytidine
lactate
oxypurinol
N-alpha-acetylarginine
1-methylguanosine
1-methyladenosine
pseudouridine
allantoin
Tryptophan
proline
N-carbamoyl-beta-alanine
glycine
TG(58:6)
1-methylguanine
citrulline
xanthine
pyridoxamine
alanine
C5-DC carnitine
PE(P-38:4)/PE(O-38:5)
N2,N2-dimethylguanosine
acetylcholine
lysine
NH4_DG(38:4)
FA 8:0;3OH
homoarginine
2-aminobutyrate
kynurenic acid
histidine
PE(P-36:4)/PE(O-36:5)
alpha-glycerophosphocholine
glycoursodeoxycholate
arginine
PE(P-38:5)/PE(O-38:6)
PE(P-34:1)/PE(O-34:2)
inositol/theophylline
proline-betaine
NH4_DG(36:0)
trigonelline
DG(36:0)
TG(46:0)
Hazard ratio
Hazard ratio
Hazard ratio

Figure S1: The association of metabolites with TBM mortality, related to Figure 1

(A) Heatmap of Spearman correlations of 469 metabolites. Each panel for each cohort. Hierarchical clustering analysis based on their Spearman correlation structure. The table summarizes the count of metabolites within each metabolite classification (HMDB: the Human Metabolome Database, Nucleic Acids Res. 2007 Jan; 35:D521-6). See also Table S8.

(B) Volcano plot showing the fold change of 469 metabolites between TBM and non-infectious control groups, with unadjusted p-values from the Wilcoxon rank sum test. Among these, 400 metabolites are significantly higher in TBM ($p < 0.05$), while 23 are significantly higher in NIC. Green dots represent the top-hit metabolites.

(C) Scatter plots of projection of patients' metabolites onto the two main principal components. The 95% confidence ellipse was plotted for diagnostic group using multivariate t-distribution ellipse. PC = principal component.

(D) Figure shows the ranking plot of 469 metabolites in the screening set, visualizing the distribution of the magnitude of metabolite-mortality associations. Each dot and its error bars represent the median rank and the 2.5% and 97.5% quantiles of the rank for each metabolite. Based on the three criteria, 107 metabolites were selected as the top-ranking metabolites in the screening set. The red dots (and error bars) correspond to harmful metabolites (i.e., HR estimate > 1), while the dark blue dots (and error bars) correspond to protective metabolites (i.e., HR estimate < 1). Metabolites not selected by the three criteria are shown in grey.

(E) The Panel 1, Panel 2 and Panel 3 corresponds to the forest plots of 107 selected metabolites as the top-ranking metabolites in the screening set (Indonesia), within cohort validation set (Indonesia) and the external validation set (Vietnam). In the three forest plots, each dot shows hazard ratio estimates (with confidence interval) of an increase of one standard deviation of abundance of the respective metabolites. Blue indicates $P<0.05$ and green $P\geq0.05$.

S2.1. Fatty Acids

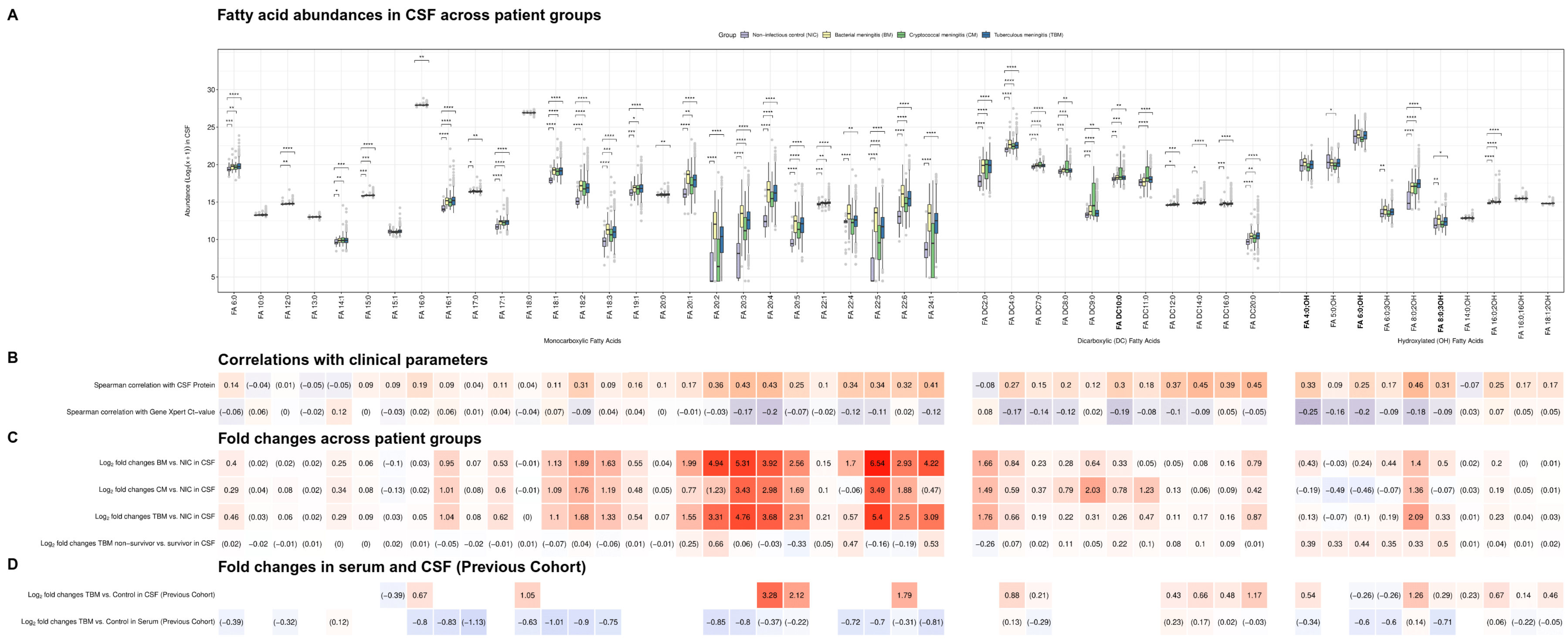

S2.2. Carnitine

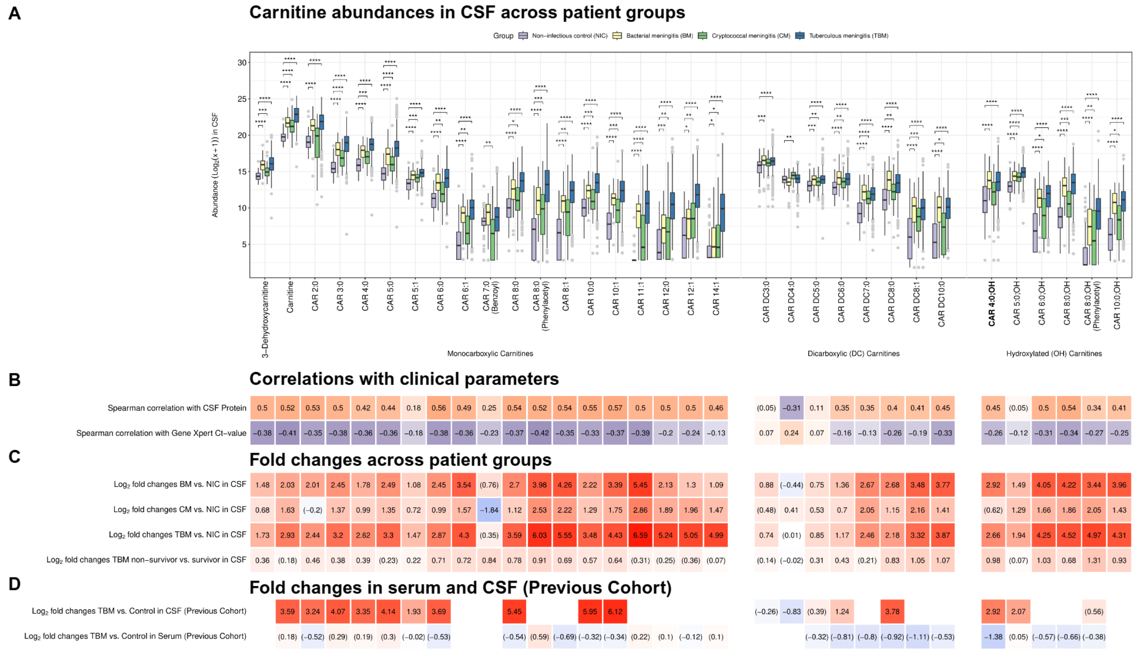

Figure S2: Relative abundance circulating fatty acids and carnitines in CSF across patient groups, related to Figure 3

(2.1) Abundances of available fatty acids in CSF and serum across patient groups and Spearman correlations with clinical parameters for TBM. Fatty acids are ordered by their length, number of double bonds, and class: mono-carboxylic, dicarboxylic (DC), and having a hydroxy (OH) group. Metabolites identified in the primary analysis are highlighted in bold. Panel A: Distributions of fatty acids where asterisks denote the significance levels following Wilcoxon statistical test (**** < $1x10^{-4}$, *** [$1x10^{-4}$, 0.001], ** [0.001, 0.01], * [0,01, 0.05]), where patient groups are compared to the non-infectious control (NIC) group. Panel B: Spearman correlations with CSF protein and bacterial load (GeneXpert Ct-value) for the TBM group, where correlations with unadjusted p-value > 0.05 are denoted by in parentheses. Panel C: Log_2 fold changes for TBM, bacterial meningitis (BM), and cryptococcal meningitis (CM) patients vs. NIC as well as non-survivors vs. survivors within TBM. Fold changes with adjusted p-value > 0.05 are enclosed in parentheses. Panel D: Log_2 fold changes for TBM patients vs. non-infectious control in CSF and serum, re-analyzed from the previous cohort (van Laarhoven et al., 2018). Fold changes with unadjusted p-value > 0.05 are enclosed in parentheses. Fold changes for fatty acids not measured in this data or not passing QC are left blank.

(2.2) Abundances of available carnitines in CSF and serum across patient groups and Spearman correlations with clinical parameters for TBM. Carnitines are ordered by their length, number of double bonds, and class: mono-carboxylic, dicarboxylic (DC), and having a hydroxy (OH) group. The analysis equals that in Figure S2.1.

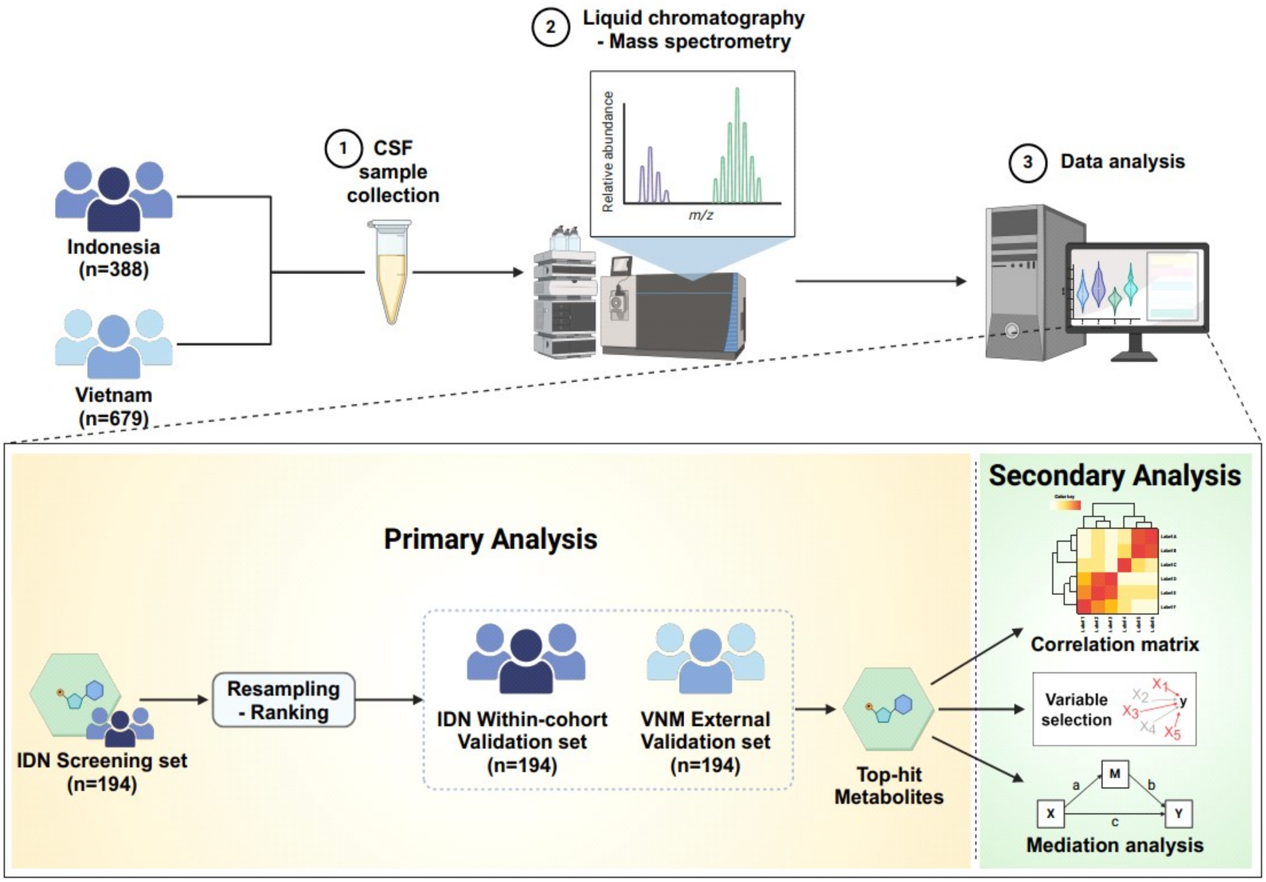

Figure S3. Analysis workflow diagram, related to STAR Methods

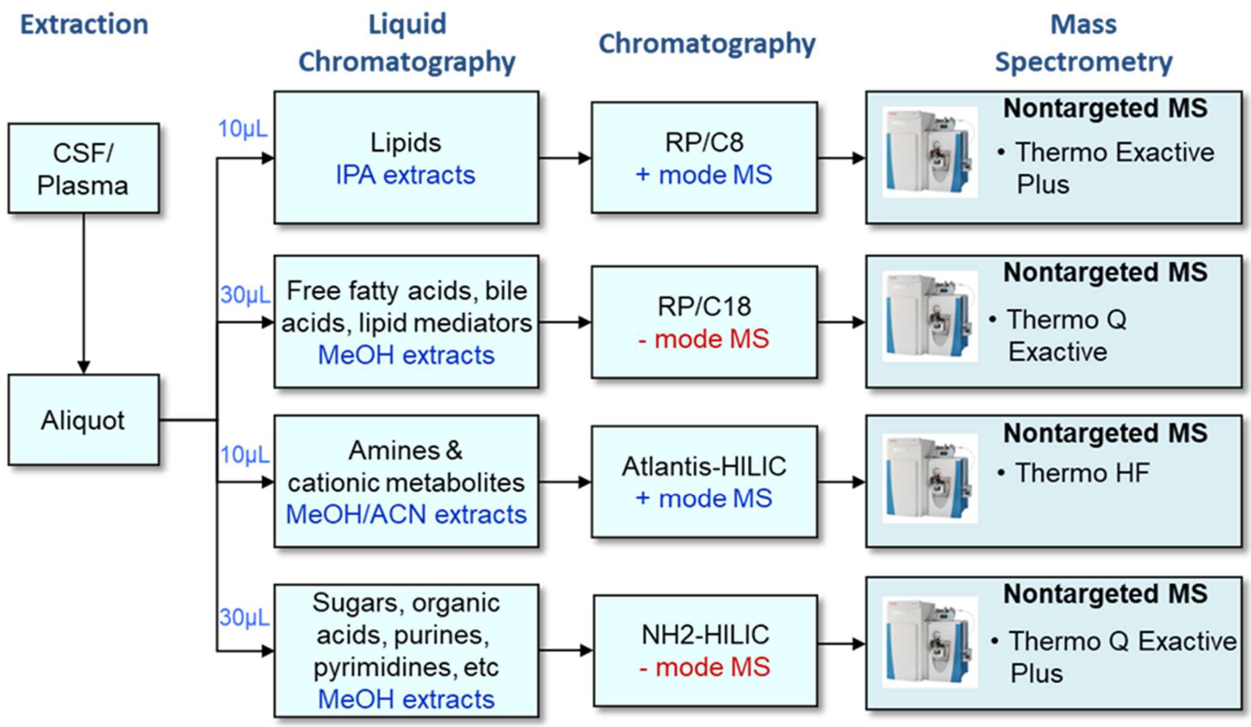

Figure S4. LC-MS-based analytical platform, related to STAR Methods

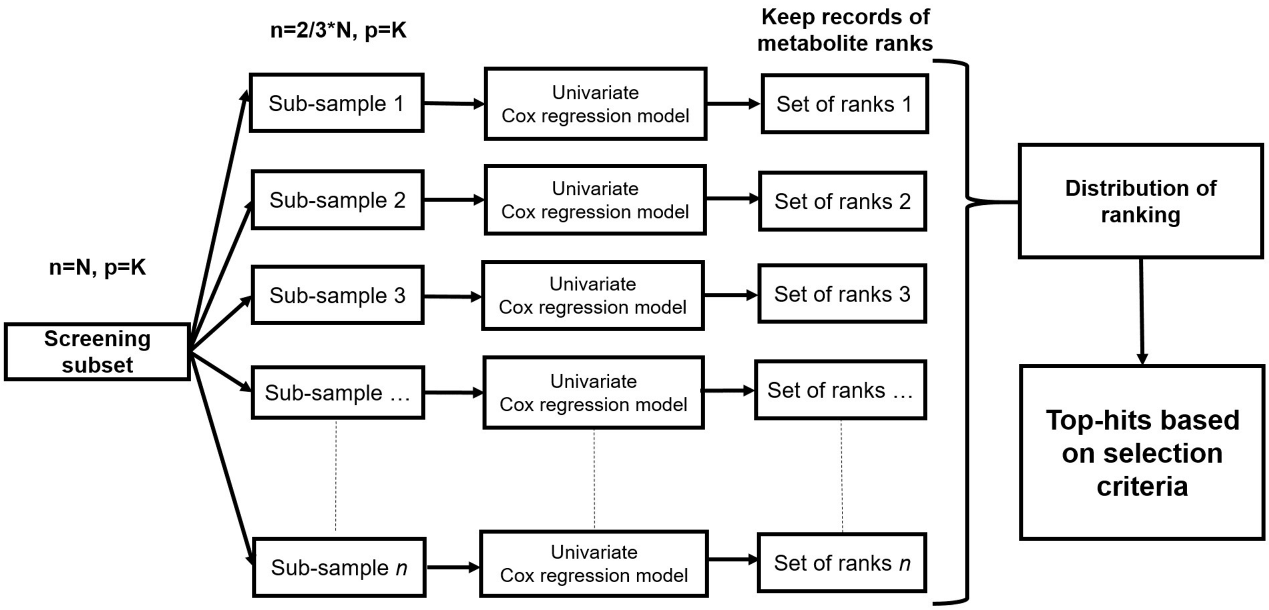

Figure S5. Sub-sampling ranking procedure, related to STAR Methods

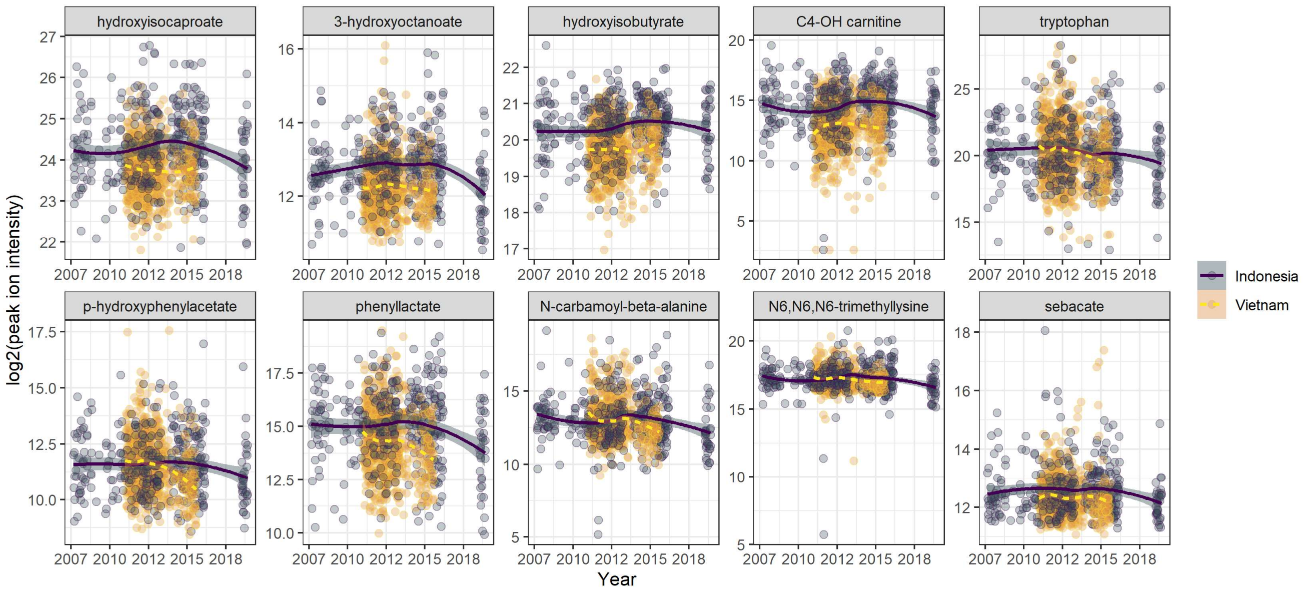

Figure S6. Top-hit metabolite's stability over time, related to STAR Methods

Table S1: Clinical characteristics association with TBM mortality-univariate analysis, related to Table 1

Characteristic	Indonesia Cohort				Vietnam Cohort			
	N	HR[1]	95% CI[1]	p-value	N	HR[1]	95% CI[1]	p-value
Sex	387				679			
Female		—	—			—	—	
Male		1.48	1.02, 2.14	0.038		1.09	0.72, 1.67	0.68
Age	387	1.03	1.01, 1.04	<0.001	679	1.02	1.00, 1.03	0.017
Diagnostic category	387				679			
Definite TBM		—	—			—	—	
Probable TBM		1.01	0.71, 1.44	0.94		1.7	1.15, 2.50	0.008
HIV status	387				679			
HIV-negative		—	—			—	—	
HIV-positive		2.32	1.44, 3.75	<0.001		2.39	1.62, 3.52	<0.001
Modified MRC disease-severity grade	368				679			
I		—	—			—	—	
II		1.84	0.74, 4.54	0.19		2.29	1.31, 4.02	0.004
III		5.25	2.05, 13.5	<0.001		6.53	3.72, 11.5	<0.001
Glasgow Coma Scale	387	0.79	0.73, 0.84	<0.001	679	0.81	0.77, 0.86	<0.001
**CSF Parameters**								
Total leukocytes — per 2-fold increase	387	0.97	0.90, 1.05	0.47	678	0.82	0.75, 0.90	<0.001
Polymorphonuclear leukocytes counts — per 2-fold increase	387	1.07	1.00, 1.15	0.048	653	0.96	0.90, 1.03	0.25
Mononuclear cells — per 2-fold increase	387	0.91	0.84, 0.99	0.024	653	0.82	0.74, 0.92	<0.001
Protein level — per 2-fold increase	387	1.12	1.01, 1.25	0.04	671	0.97	0.80, 1.17	0.71
CSF to blood glucose ratio	387	0.23	0.07, 0.77	0.018	671	0.81	0.30, 2.21	0.69
Mean GeneXpert Ct value	126	0.99	0.95, 1.02	0.45	654	1	0.97, 1.03	0.83

[1]HR = Hazard Ratio, CI = Confidence Interval

Table S2: Patient's characteristics of TBM in compared with other meningitis, related to Table 1

Characteristics	n[1]	Bacterial meningitis, N = 50[1,2]	n[1]	Cryptococcal meningitis, N = 62[1,2]	n[1]	Non-infectious control, N = 54[1,2]	n[1]	Tuberculous meningitis, N = 1,067[1,2]	p-value[3]
Female sex — no. (%)	20	8 (40%)	35	7 (20%)	54	24 (44%)	1067	369 (65%)	0.12
Age — median (1st and 3rd interquartile) (years)	20	46 (34, 57)	56	33 (27, 37)	54	35 (25, 44)	1067	34 (27, 44)	0.012
HIV-positive — no. (%)	50	0 (0%)	61	60 (98%)	54	11 (20%)	1067	284 (27%)	<0.001
**CSF parameter**									
Total leukocytes count — cells/μL	35	1,900 (739, 5,460)	35	103 (28, 201)	54	2 (1, 3)	1066	150 (49, 336)	<0.001
Polymorphonuclear leukocytes counts— cells/μL	34	1,527 (538, 4,986)	35	17 (6, 97)	46	1 (0, 1)	1041	22 (3, 100)	<0.001
Mononuclear cells — cells/μL	34	307 (134, 646)	35	36 (7, 89)	46	2 (1, 3)	1041	98 (38, 206)	<0.001
Protein level, g/L	35	190 (110, 380)	35	76 (58, 167)	54	40 (26, 59)	1059	145 (90, 240)	<0.001
CSF to blood glucose ratio	32	0.30 (0.10, 0.47)	35	0.30 (0.20, 0.40)	54	0.60 (0.56, 0.70)	1059	0.28 (0.17, 0.40)	<0.001
Median Glasgow coma scale (1st and 3rd interquartile)	20	13 (9, 14)	35	15 (13, 15)	54	15 (12, 15)	1067	14 (12, 15)	0.01

[1]Number of non-missing observations; median (1st and 3rd interquartile) for continuous variables and frequency (%) for categorical variables.
[2]Patients lost to follow-up included 7 with BM, 12 with CM, 21 in the Non-infectious control group, and 12 with TBM. Additionally, 30 BM patients and 27 CM patients did not have follow-up.
[3]Pearson's chi-squared test for category variables; Wilcoxon rank sum test for continuous variables.

Table S3: Association of top-hit metabolites with 6-month mortality in both cohorts, related to Table 2

Metabolite	Indonesia Cohort				Vietnam Cohort			
	N	HR[1]	95% CI[1]	p-value	N	HR[1]	95% CI[1]	p-value
FA 6:0;OH	387	1.46	1.26, 1.69	<0.001	679	1.48	1.21, 1.82	<0.001
FA 8:0;3OH	385	1.35	1.17, 1.57	<0.001	678	1.63	1.41, 1.90	<0.001
FA 4:0;OH	387	1.44	1.21, 1.72	<0.001	679	1.37	1.12, 1.66	0.002
CAR 4:0;OH	386	1.37	1.12, 1.67	0.003	679	1.46	1.17, 1.82	<0.001
Tryptophan	386	1.34	1.16, 1.55	<0.001	679	1.56	1.34, 1.82	<0.001
p-Hydroxyphenylacetate	385	1.55	1.33, 1.81	<0.001	678	1.44	1.24, 1.68	<0.001
Phenyllactate	385	1.54	1.28, 1.85	<0.001	678	1.71	1.43, 2.06	<0.001
N-carbamoyl-beta-alanine	386	1.43	1.25, 1.65	<0.001	679	1.63	1.40, 1.89	<0.001
N6,N6,N6-Trimethyllysine	386	1.77	1.51, 2.08	<0.001	679	1.44	1.19, 1.75	<0.001
Sebacate	387	1.23	1.09, 1.39	<0.001	679	1.33	1.16, 1.52	<0.001

[1]HR = Hazard Ratio, CI = Confidence Interval

Table S4: Pathway enrichment analysis results for day 60 mortality, related to Figure 1 and Table 2

Pathway	Total Cmpd	Hits	Statistic Q	Expected Q	Raw p	FDR
Pyrimidine metabolism	39	6	30,521	0.0943	7.85e-13	4.79e-10
Tryptophan metabolism	41	5	2,013	0.0943	4.18e-08	1.27e-06
Glycerolipid metabolism	16	2	23,571	0.0943	1.03e-05	0.0002
Sphingolipid metabolism	32	4	25,504	0.0943	1.08e-05	0.0002
Tyrosine metabolism	42	5	18,077	0.0943	3.16e-08	0.0039
Galactose metabolism	27	4	12,544	0.0943	0.0062	0.0628
Ascorbate and aldarate metabolism	9	2	17,397	0.0943	2.13e-06	0.1624
Inositol phosphate metabolism	30	2	17,397	0.0943	2.13e-06	0.1624
Pantothenate and CoA biosynthesis	20	6	11,218	0.0943	0.0345	0.2304
Pyruvate metabolism	23	2	1,313	0.0943	0.0378	0.2304
Pentose and glucuronate interconversions	19	1	18,944	0.0943	0.0675	0.3742
Glycolysis / Gluconeogenesis	26	1	1,823	0.0943	0.1010	0.5132
Neomycin, kanamycin and gentamicin biosynthesis	2	1	17,233	0.0943	0.1774	0.8326
beta-Alanine metabolism	21	4	10,942	0.0943	0.1995	0.8694
Citrate cycle (TCA cycle)	20	5	0.7842	0.0943	0.2581	0.0001
Phenylalanine metabolism	8	2	14,043	0.0943	0.6173	0.0002
Phenylalanine, tyrosine and tryptophan biosynthesis	4	2	14,043	0.0943	0.6173	0.0002
Valine, leucine and isoleucine degradation	39	6	11,003	0.0943	0.0001	0.0004
Purine metabolism	70	9	0.5145	0.0943	0.0001	0.0004
Steroid hormone biosynthesis	87	4	0.8380	0.0943	0.0002	0.0006
Valine, leucine and isoleucine biosynthesis	8	6	10,457	0.0943	0.0002	0.0007
Lipoic acid metabolism	28	2	0.7638	0.0943	0.0013	0.0036
Pentose phosphate pathway	23	1	0.9059	0.0943	0.0019	0.0048
Lysine degradation	30	3	0.7645	0.0943	0.0019	0.0048
Starch and sucrose metabolism	18	3	0.6969	0.0943	0.0025	0.0060
Caffeine metabolism	10	1	0.8553	0.0943	0.0026	0.0060
Primary bile acid biosynthesis	46	7	0.6702	0.0943	0.0029	0.0065
Ubiquinone and other terpenoid-quinone biosynthesis	18	1	0.8082	0.0943	0.0034	0.0073
Glycerophospholipid metabolism	36	5	0.5343	0.0943	0.0042	0.0089
Amino sugar and nucleotide sugar metabolism	42	1	0.7393	0.0943	0.0050	0.0103
Glycine, serine and threonine metabolism	33	10	0.4562	0.0943	0.0072	0.0141
Taurine and hypotaurine metabolism	8	2	0.5028	0.0943	0.0171	0.0325
Histidine metabolism	16	6	0.2961	0.0943	0.0204	0.0378
Cysteine and methionine metabolism	33	5	0.3306	0.0943	0.0255	0.0457
Glyoxylate and dicarboxylate metabolism	31	8	0.2977	0.0943	0.0279	0.0487
Butanoate metabolism	15	3	0.3390	0.0943	0.0356	0.0603
Thiamine metabolism	7	1	0.4048	0.0943	0.0382	0.0629

Table S5: Metabolites associated with mortality, related to Figure 1 and Table 2

Metabolite	Description	Biological effect and related metabolic pathways
FA 6:0;OH<br>(hydroxy-isocaproate)<br>$C_6H_{12}O_3$	Hydroxy-carboxylic acid	• Beta-oxidation intermediate
FA 8:0;3OH<br>(3-hydroxyoctanoate)<br>$C_8H_{16}O_3$	Hydroxy-carboxylic acid	• Beta-oxidation intermediate<br>• Ligand for GPR109B (HCA3), a GPCR expressed in adipocytes and immune cells (Ahmed et al., 2009)<br>• HCA3 ligands suppress cytokine expression (Mandrika et al., 2018)
FA 4:0;OH<br>(alpha-hydroxybutyrate or beta-hydroxybutyrate)<br>$C_4H_8O_3$	Hydroxy-carboxylic acid	• Beta-oxidation intermediate<br>• Ketone body
CAR 4:0;OH<br>(3-hydroxybutyrylcarnitine)<br>$C_{11}H_{21}NO_5$	Conjugate of FA 4:0;OH and carnitine	• Beta-oxidation ketone body metabolite<br>• Elevated in plasma in response to starvation (Steinhauser et al., 2018)
Tryptophan<br>$C_{11}H_{12}N_2O_2$	Amino acid	• Precursor of serotonin and kynurenine
p-Hydroxyphenylacetate<br>$C_8H_8O_3$		• Downstream metabolites of the transamination product of phenylalanine, phenylpyruvate (Antoshechkin et al., 1991; van Spronsen et al., 2021)
Phenyllactate<br>$C_9H_{10}O_3$	Phenylalanine catabolite; produced from phenylpyruvate via the action of lactate dehydrogenase	• Antimicrobial compound synthesized by lactic acid bacteria<br>• Decreases ROS production in mitochondria and neutrophils (Beloborodova et al., 2012)<br>• Downstream metabolites of the transamination product of phenylalanine, phenylpyruvate (Antoshechkin et al., 1991; van Spronsen et al., 2021)
N-carbamoyl-beta-alanine<br>(3-Ureidopropionic acid)<br>$C_4H_8N_2O_3$	An intermediate in the metabolism of uracil	• Inhibits respiratory chain complex V and may function as an endogenous neurotoxin in the context of 3-ureidopropionase deficiency (Kölker et al., 2001)<br>• Higher plasma levels linked to heart failure risk (Yazdani et al., 2024) and frailty (Marron et al., 2019)
Sebacic acid<br>(sebacate)<br>$C_{10}H_{18}O_4$	Dicarboxylic acid	• Elevated in urine of patients with medium-chain acyl-CoA dehydrogenase deficiency (Gregersen et al., 1983)
N6,N6,N6-trimethyllysine<br>$C_9H_{20}N_2O_2$	Methylated derivative of lysine	• Precursor for carnitine biosynthesis

Table S6: Distribution of log2-transformed peak ion abundances for the metabolites associated to outcome, stratified by TBM severity (MRC grade), related to Figure 2, and stratified by HIV groups, related to Figure 4

**Metabolites**	**TBM severity (MRC grade)**				**HIV Groups**		
	**Grade I**, N = 287[1]	**Grade II**, N = 582[1]	**Grade III**, N = 179[1]	**p-value**[2]	**HIV-negative**, N = 783[1]	**HIV-positive**, N = 284[1]	**p-value**[3]
FA 6:0;OH	28.88 (28.41, 29.39)	29.34 (28.73, 30.01)	29.55 (29.11, 30.14)	<0.0001	29.33 (28.76, 30.02)	28.99 (28.46, 29.52)	<0.001
FA 8:0;3OH	14.62 (14.06, 15.11)	15.10 (14.45, 15.74)	15.14 (14.53, 15.67)	<0.0001	15.02 (14.37, 15.69)	14.76 (14.32, 15.38)	0.004
FA 4:0;OH	25.11 (24.52, 25.61)	25.76 (25.11, 26.39)	25.98 (25.35, 26.47)	<0.0001	25.72 (25.01, 26.40)	25.35 (24.72, 25.90)	<0.001
CAR 4:0;OH	5.29 (4.75, 5.79)	5.85 (5.37, 6.41)	5.93 (5.36, 6.48)	<0.0001	5.80 (5.27, 6.41)	5.53 (4.96, 6.01)	<0.001
Tryptophan	8.58 (7.96, 9.41)	8.73 (8.17, 9.54)	9.05 (8.38, 9.49)	0.0003	8.69 (8.08, 9.42)	8.92 (8.31, 9.67)	0.001
p-hydroxyphenylacetate	8.34 (7.84, 8.98)	8.53 (7.96, 9.30)	8.97 (8.30, 9.68)	<0.0001	8.50 (7.93, 9.24)	8.69 (8.07, 9.28)	0.077
Phenyllactate	7.88 (7.24, 8.42)	8.32 (7.68, 8.89)	8.66 (8.08, 9.17)	<0.0001	8.25 (7.60, 8.84)	8.33 (7.58, 8.86)	0.6
N-carbamoyl-beta-alanine	8.51 (8.13, 8.94)	8.58 (8.07, 9.16)	8.84 (8.25, 9.71)	0.001	8.50 (8.05, 9.07)	8.83 (8.36, 9.50)	<0.001
N6,N6,N6-Trimethyllysine	20.29 (19.91, 20.65)	20.45 (19.95, 20.98)	20.67 (20.09, 21.29)	<0.0001	20.42 (19.95, 20.96)	20.38 (19.92, 20.86)	0.3
Sebacate	31.26 (30.91, 31.63)	31.35 (30.96, 31.85)	31.44 (31.07, 31.96)	0.0001	31.32 (30.95, 31.83)	31.40 (31.05, 31.79)	0.4

[1]*Median (1st, 3rd interquartile)*
[2]*p-value derived from trend test (Jonckheere-Terpstra test)*
[3]*p-value derived from Wilcoxon rank sum test*

Table S7: Causal mediation analysis of the impact of the pre-treatment metabolites to mortality by TBM severity, related to Figure 2

Metabolite	Total impact			Natural Direct Impact			Natural Indirect Impact			Proportion Mediated		
	HR[1]	95% CI[1]	p-value	HR[1]	95% CI[1]	p-value	HR[1]	95% CI[1]	p-value	HR[1]	95% CI[1]	p-value
FA 6:0;OH	1.67	1.42; 1.95	<0.001	1.67	1.42; 1.95	<0.001	1	1.00; 1.00	1	0	0.00; 0.00	1
FA 8:0;3OH	1.59	1.39; 1.84	<0.001	1.57	1.37; 1.82	<0.001	1.01	1.00; 1.04	0.061	0.04	0.00; 0.1	0.064
FA 4:0;OH	1.59	1.36; 1.89	<0.001	1.59	1.36; 1.89	<0.001	1	1.00; 1.00	1	0	0.00; 0.00	1
CAR 4:0;OH	1.77	1.46; 2.2	<0.001	1.62	1.35; 2.01	<0.001	1.09	1.04; 1.17	<0.001	0.19	0.09; 0.33	<0.001
Tryptophan	1.48	1.3; 1.69	<0.001	1.37	1.22; 1.57	<0.001	1.08	1.03; 1.12	<0.001	0.21	0.09; 0.33	0.001
p-Hydroxyphenylacetate	1.41	1.21; 1.65	<0.001	1.31	1.14; 1.53	<0.001	1.07	1.03; 1.13	<0.001	0.23	0.10; 0.39	<0.001
Phenyllactate	1.61	1.34; 1.94	<0.001	1.54	1.29; 1.87	<0.001	1.04	1.01; 1.09	<0.001	0.11	0.03; 0.21	<0.001
N-carbamoyl-beta-alanine	1.6	1.36; 1.86	<0.001	1.48	1.28; 1.72	<0.001	1.08	1.03; 1.12	<0.001	0.19	0.07; 0.29	0.001
N6,N6,N6-Trimethyllysine	1.56	1.32; 1.83	<0.001	1.55	1.32; 1.83	<0.001	1	1.00; 1.01	0.444	0.01	-0.01; 0.03	0.454
Sebacate	1.26	1.14; 1.45	<0.001	1.26	1.14; 1.45	<0.001	1	1.00; 1.01	0.589	-0.01	-0.02; 0.05	0.609

[1]HR = Hazard Ratio, CI = Confidence Interval

Supplemental references

1. van Laarhoven, A. et al. Cerebral tryptophan metabolism and outcome of tuberculous meningitis: an observational cohort study. Lancet Infect. Dis. 18, 526–535 (2018).

2. Ahmed, K., Tunaru, S., Langhans, C.-D., Hanson, J., Michalski, C. W., Kölker, S., Jones, P. M., Okun, J. G., & Offermanns, S. (2009). Deorphanization of GPR109B as a receptor for the beta-oxidation intermediate 3-OH-octanoic acid and its role in the regulation of lipolysis. The Journal of Biological Chemistry, 284(33), 21928–21933. https://doi.org/10.1074/jbc.M109.019455

3. Antoshechkin, A. G., Chentsova, T. V, VYu, T., Naritsin, D. B., & Railian, G. P. (1991). Content of phenylalanine, tyrosine and their metabolites in CSF in phenylketonuria. Journal of Inherited Metabolic Disease, 14(5), 749–754. https://doi.org/10.1007/BF01799945

4. Beloborodova, N., Bairamov, I., Olenin, A., Shubina, V., Teplova, V., & Fedotcheva, N. (2012). Effect of phenolic acids of microbial origin on production of reactive oxygen species in mitochondria and neutrophils. Journal of Biomedical Science, 19(1), 89. https://doi.org/10.1186/1423-0127-19-89

5. Gregersen, N., Kølvraa, S., Rasmussen, K., Mortensen, P. B., Divry, P., David, M., & Hobolth, N. (1983). General (medium-chain) acyl-CoA dehydrogenase deficiency (non-ketotic dicarboxylic aciduria): quantitative urinary excretion pattern of 23 biologically significant organic acids in three cases. Clinica Chimica Acta; International Journal of Clinical Chemistry, 132(2), 181–191. https://doi.org/10.1016/0009-8981(83)90246-2

6. Kölker, S., Okun, J. G., Hörster, F., Assmann, B., Ahlemeyer, B., Kohlmüller, D., Exner-Camps, S., Mayatepek, E., Krieglstein, J., & Hoffmann, G. F. (2001). 3-Ureidopropionate contributes to the neuropathology of 3-ureidopropionase deficiency and severe propionic aciduria: A Hypothesis. Journal of Neuroscience Research, 66(4), 666–673. https://doi.org/10.1002/jnr.10012

7. Mandrika, I., Tilgase, A., Petrovska, R., & Klovins, J. (2018). Hydroxycarboxylic Acid Receptor Ligands Modulate Proinflammatory Cytokine Expression in Human Macrophages and Adipocytes without Affecting Adipose Differentiation. Biological & Pharmaceutical Bulletin, 41(10), 1574–1580. https://doi.org/10.1248/bpb.b18-00301

8. Marron, M. M., Harris, T. B., Boudreau, R. M., Clish, C. B., Moore, S. C., Murphy, R. A., Murthy, V. L., Sanders, J. L., Shah, R. V, Tseng, G. C., Wendell, S. G., Zmuda, J. M., & Newman, A. B. (2019). Metabolites Associated with Vigor to Frailty Among Community-Dwelling Older Black Men. Metabolites, 9(5). https://doi.org/10.3390/metabo9050083

9. Steinhauser, M. L., Olenchock, B. A., O'Keefe, J., Lun, M., Pierce, K. A., Lee, H., Pantano, L., Klibanski, A., Shulman, G. I., Clish, C. B., & Fazeli, P. K. (2018). The circulating metabolome of human starvation. JCI Insight, 3(16). https://doi.org/10.1172/jci.insight.121434

10. van Spronsen, F. J., Blau, N., Harding, C., Burlina, A., Longo, N., & Bosch, A. M. (2021). Phenylketonuria. Nature Reviews. Disease Primers, 7(1), 36. https://doi.org/10.1038/s41572-021-00267-0

11. Yazdani, A., Mendez-Giraldez, R., Yazdani, A., Wang, R.-S., Schaid, D. J., Kong, S. W., Hadi, M. R., Samiei, A., Samiei, E., Wittenbecher, C., Lasky-Su, J., Clish, C. B., Muehlschlegel, J. D., Marotta, F., Loscalzo, J., Mora, S., Chasman, D. I., Larson, M. G., & Elsea, S. H. (2024). Broadcasters, receivers, functional groups of metabolites, and the link to heart failure by revealing metabolomic network connectivity. Metabolomics : Official Journal of the Metabolomic Society, 20(4), 71. https://doi.org/10.1007/s11306-024-02141-y
