# Supplementary material for: Pre-treatment untargeted cerebrospinal fluid metabolomic profiling in tuberculous meningitis uncovers pathways associated with mortality
Source: Med. Author manuscript; Available in PMC 2026 Feb 19. (PMC7618751; doi:10.1016/j.medj.2025.100703)
Supplement: Supplementary Materials [file EMS212236-supplement-Supplementary_Materials.zip › 1-s2.0-S2666634025001308-mmc3.pdf]

Med

Article

Pre-treatment untargeted cerebrospinal fluid metabolomic profiling in tuberculous meningitis uncovers pathways associated with mortality

Graphical abstract

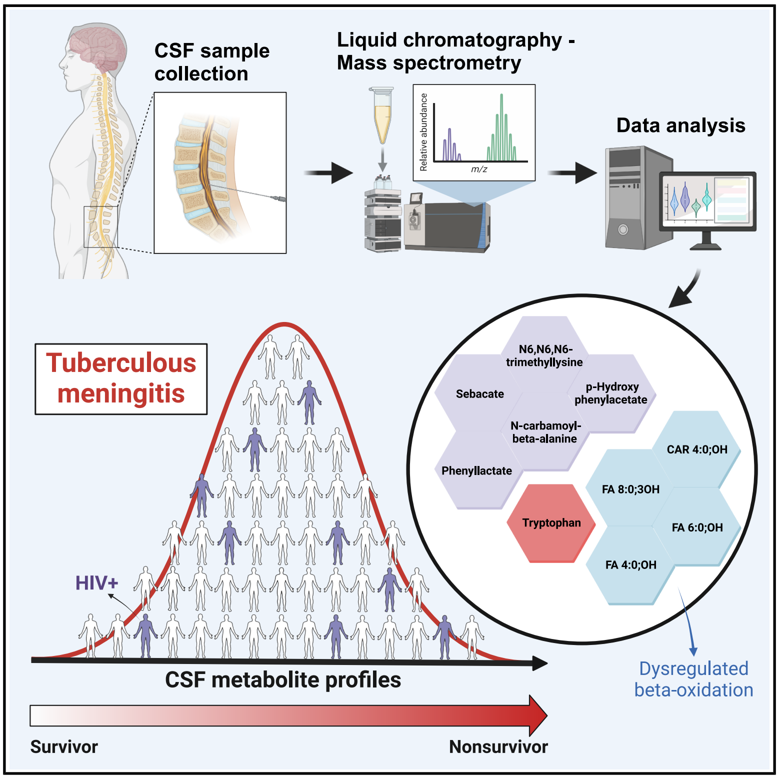

Highlights

- Analysis of CSF metabolites from 1,067 TBM patients using untargeted metabolomics
- Nine metabolites linked to TBM mortality, independent of HIV, severity, tryptophan
- The results show altered fatty acid β-oxidation strongly linked to TBM-related mortality

Authors

Thanh Hoang Nhat Le, Kirsten C. J. van Abeelen, Edwin Ardiansyah, ..., Clary B. Clish, Nguyen Thuy Thuong Thuong, Arjan van Laarhoven

Correspondence

thuongnttt@oucru.org (N.T.T.T.), arjan.vanlaarhoven@radboudumc.nl (A.v.L.)

In brief

Le et al. used untargeted metabolomics to study pre-treatment cerebrospinal fluid (CSF) samples of 1,067 tuberculous meningitis patients from Vietnam and Indonesia. Sixty-day mortality was high (21.6%) and was robustly associated with increased CSF hydroxylated fatty acids. Dysregulated β-oxidation may be an important and potentially amendable process in tuberculous meningitis mortality.

Translation to Patients

Le et al., 2025, Med *6*, 100703
September 12, 2025 © 2025 The Authors.
Published by Elsevier Inc.
https://doi.org/10.1016/j.medj.2025.100703

CellPress

Med

CellPress
OPEN ACCESS

Article

Pre-treatment untargeted cerebrospinal fluid metabolomic profiling in tuberculous meningitis uncovers pathways associated with mortality

Thanh Hoang Nhat Le,[1] Kirsten C.J. van Abeelen,[2] Edwin Ardiansyah,[2,3] Julian Avila-Pacheco,[4] Sofiati Dian,[3] Gesa Carstens,[2] Lara Schramke,[2] Hoang Thanh Hai,[1] Tran Binh Minh Nguyen,[1] Thai Minh Triet,[1] Amy Deik,[4] Jesse Krejci,[4] Jeff Pruyne,[4] Lucas Dailey,[4] Bachti Alisjahbana,[3] Mihai G. Netea,[2] Riwanti Estiasari,[6] Trinh Thi Bich Tram,[1] Joseph Donovan,[1] Dorothee Heemskerk,[1] Thi Hong Chau Tran,[1] Nguyen Duc Bang,[5] Ahmad Rizal Ganiem,[3] Raph L. Hamers,[6] Rovina Ruslami,[3] Darma Imran,[6] Kartika Maharani,[6] Vinod Kumar,[2] Reinout van Crevel,[2,7] Guy Thwaites,[1,7] Clary B. Clish,[4] Nguyen Thuy Thuong Thuong,[1,7,8,9,*] and Arjan van Laarhoven[2,7,8,*]

[1]Oxford University Clinical Research Unit, Ho Chi Minh City, Vietnam
[2]Department of Internal Medicine and Radboud Community for Infectious Diseases, Radboud University Medical Center, Nijmegen, the Netherlands
[3]Research Center for Care and Control of Infectious Diseases, Universitas Padjadjaran, Bandung, Indonesia
[4]Broad Institute of MIT and Harvard, Cambridge, MA, USA
[5]Pham Ngoc Thach Hospital, Ho Chi Minh City, Vietnam
[6]Oxford University Clinical Research Unit Indonesia, Faculty of Medicine, Universitas Indonesia, Jakarta, Indonesia
[7]Centre for Tropical Medicine and Global Health, Nuffield Department of Medicine, University of Oxford, Oxford, UK
[8]These authors contributed equally
[9]Lead contact
*Correspondence: thuongnttt@oucru.org (N.T.T.T.), arjan.vanlaarhoven@radboudumc.nl (A.v.L.)
https://doi.org/10.1016/j.medj.2025.100703

CONTEXT AND SIGNIFICANCE Meningitis caused by tuberculosis is a medical emergency. Immunopathology plays an important role, but current host-directed therapy is insufficient. Corticosteroids reduce mortality, but still half of these patients die or remain disabled. CSF tryptophan has been confirmed as a determinant of tuberculous meningitis mortality, and Le et al. hypothesized that metabolites from other pathways may also be important. Pre-treatment CSF samples from 1,067 tuberculous meningitis patients with and without HIV were measured by untargeted metabolomics, which identified 469 metabolites. Nine new metabolites, including three hydroxylated fatty acids with a maximum carbon length of 8, were identified and validated to predict TBM mortality independent of HIV status. This suggests dysregulated cerebral β-oxidation as an important and potential modifiable determinant of mortality.

SUMMARY

Background: Dysregulation of cerebrospinal fluid (CSF) tryptophan metabolism contributes to the high mortality of tuberculous meningitis (TBM). We aimed to identify novel metabolic pathways associated with TBM mortality through untargeted metabolome-wide analysis.
Methods: We measured 619 metabolites using untargeted liquid chromatography-mass spectrometry in pre-treatment CSF from adults with TBM from Indonesia (n = 388, 34 HIV positive) and Vietnam (n = 679, 250 HIV positive). Sixty-day mortality was modeled using Cox regression, adjusting for age and HIV status. Metabolites were ranked in a screening subset (n = 194, Indonesia) and validated in the same cohort (n = 194) and externally (n = 679, Vietnam). Secondary analysis included variable selection, clustering to classify associated metabolites into subgroups, comparison with non-infectious controls, and correlation with patient characteristics, CSF cytokines, CSF protein, and serum metabolite concentrations.
Findings: Sixty-day mortality was 21.6% and was associated with the concentration of 10 CSF metabolites, including tryptophan. The strongest association was with 3-hydroxyoctanoate (FA 8:0;3OH), part of a cluster of hydroxylated fatty acids also including hydroxy-isocaproate (FA 6:0;OH), hydroxyisobutyrate (FA 4:0;OH), and C4-OH-carnitine. These fatty acids correlated weakly with CSF tumor necrosis factor alpha, interleukin-6 (IL-6), leukocyte counts, bacterial load, and CSF protein. Mediation analysis showed that the variation in fatty acids was linked directly to mortality rather than through disease severity.

© 2025 The Authors. Published by Elsevier Inc.
This is an open access article under the CC BY license (http://creativecommons.org/licenses/by/4.0/).

Conclusion: We identified and validated nine new metabolites associated with TBM mortality, independent of HIV status, disease severity, and tryptophan. These metabolites suggest that altered fatty acid β-oxidation is linked to TBM-associated mortality. Interventions targeting cerebral fatty acid metabolism may improve survival of TBM.

Funding: National Institute of Health; Wellcome Trust, UK.

INTRODUCTION

Tuberculous meningitis (TBM) is the most severe form of tuberculosis (TB), resulting in high mortality and long-term disability.[1] In 2019, an estimated 164,000 adults developed TBM globally, with around 25% of these cases occurring in people living with HIV.[2] TBM is caused by *Mycobacterium tuberculosis* invading the brain and meninges, leading to an inflammatory response.[3,4] A high bacterial load predicts neurological events,[4] while increased brain injury markers predict mortality.[5]

The role of the inflammatory response in determining TBM outcome remains incompletely understood. Low total cerebrospinal fluid (CSF) leukocyte counts and low CSF cytokines have been linked to mortality,[4] and high neutrophil counts have been associated with neurological complications[4] and mortality.[6] Most importantly, adjunctive corticosteroid treatment improves survival from TBM in HIV-negative patients.[7] However, this benefit remains unproven in HIV-positive adults with TBM.[8] Adding aspirin to corticosteroids showed an aspirin dose-dependent inhibition of thromboxane A2 and upregulation of pro-resolving CSF protectins, resulting in a potential reduction of new infarcts and deaths in TBM patients.[9] Further study of CSF lipid mediators showed that severe TBM was associated with an increase in prostaglandins and leukotrienes, while survivors had a significant increase in some pro-resolving mediators.[10]

Leukocyte cellular metabolism is important for mycobacterial killing and the subsequent inflammatory response.[11] Cerebral metabolism may also be relevant in the outcome of central nervous system infections. Increased energy demand may cause reductions in the usual energy supplies, including glucose, lactate, and ketone bodies for neurons and fatty acids for ketone bodies (refer to Box 1 for background). Previous research has linked cerebral tryptophan metabolism to mortality in HIV-negative and HIV-positive adults with TBM.[12,13] Yet, a significant portion of TBM mortality variation remains unexplained, suggesting the involvement of other metabolites besides tryptophan.

Therefore, we aimed to identify additional metabolites associated with mortality in TBM, using an untargeted metabolomic approach in HIV-negative and HIV-positive adults from Indonesia and Vietnam. To identify robust signals in the high-dimensional untargeted metabolites data, we used a two-step approach. We first ranked metabolites based on their association with mortality in Indonesian TBM patients and then validated these associations both within the Indonesian cohort and externally in an independent Vietnamese cohort. By integrating these validated findings with clinical characteristics, we aimed to improve our understanding of TBM pathogenesis and the causes of mortality and to identify potential targets for host-directed therapy.

RESULTS

Patient characteristics and association with mortality

Patients with TBM from Indonesia were younger than those from Vietnam (median age [first, third interquartile]: 30 [23, 38] vs. 36 [29, 47], $p < 0.001$; Table 1). They also presented with more severe disease (92% vs. 62% with Medical Research Council [MRC] grade II and III, respectively, $p < 0.001$; see Table S1 for other clinical predictors of outcome) and had a higher day 60 mortality rate (33% vs. 15%, respectively; $p < 0.001$). The Indonesian cohort included fewer HIV-coinfected TBM patients than the Vietnamese cohort (9% vs. 37%, $p < 0.001$). CSF from patients in Indonesia had higher polymorphonuclear leukocyte counts and protein levels, lower CSF-to-blood glucose ratio, and higher bacterial loads. We also included non-infectious controls ($n = 54$) and patients with bacterial meningitis ($n = 50$) and cryptococcal meningitis ($n = 62$) who had an age range and gender distribution similar to TBM patients (Table S2).

Of 619 measured metabolites, 469 metabolites had a coefficient of variation below 30% and were detected in at least 75% of patients and therefore passed quality control. More than half of these metabolites, including a large cluster of fatty acids, were strongly intercorrelated in both cohorts (Figure S1A). Of 469 metabolites, only 36 were lower, but 433 were higher in TBM patients than non-infectious controls (Figure S1B). Principal-component analysis revealed a gradient along the major principal component of metabolites profiles (PC1, accounting for 41% of the variance) from non-infectious controls to cryptococcal to bacterial to TBM, with large within-group variation (Figure S1C).

To identify robust signals in the high-dimensional untargeted metabolite data, we employed a two-step strategy. First, we used a resampling method to rank metabolites based on their association with mortality in a training set comprising of half of the Indonesian TBM patients, referred to as the screening cohort ($n = 194$ with 63 deaths). We applied 10,000 resampling-ranking iterations, each of which including two-thirds of the screening cohort for sampling and Cox regression for day 60 mortality, corrected for age and HIV. Ranked over those 10,000 iterations, we selected metabolites that showed a consistent direction of association with mortality, with the 2.5% quantile of their rank distribution falling below a pre-specified lower threshold (R1 = 75) and the 97.5% quantile below an upper threshold (R2 = 225). We thereby identified 101 metabolites associated with increased mortality and six with decreased mortality in the Indonesian screening cohort (Figure S1D).

Second, we validated these associations both internally in the remaining half of the Indonesian cohort, referred to as the within-cohort validation set ($n = 194$ with 64 deaths) or test set, and externally in an independent cohort from Vietnam ($n = 679$ with 103

CellPress
OPEN ACCESS

Box 1. Role of carnitines and FAs in brain neuropathology

Cells use FAs to generate energy through mitochondrial β-oxidation, a process that requires the reversible esterification into acyl-carnitines for transport into the mitochondria. In the brain, β-oxidation is mostly restricted to astrocytes, while neurons almost exclusively generate energy by oxidizing lactate or glucose or by ketone bodies.[14,15] This metabolic compartmentalization protects neurons, a highly metabolically active cell type, from higher levels of reactive oxygen species (ROS) produced during β-oxidation.[16] While β-oxidation and ROS are regulators of normal neuronal development, neurodegenerative pathology has been associated with dysfunctional brain β-oxidation and FA accumulation.[17,18] Astrocyte β-oxidization can help neurons cope with high FA levels while supplementing lactate and ketone bodies in a glucose-deprived FA-rich environment like the one observed during TBM.[19–21]

deaths). Adjusting for false discovery rate among these 107 metabolites, five metabolites replicated in the within-cohort validation and eight in the Vietnamese external validation (Table 2; Figure S1E). This included the replication of the previously reported association of higher CSF tryptophan levels with increased mortality,[12] confirmed using a tryptophan metabolite-specific profiling method.[13] Additionally, the metabolites associated with increased mortality were hydroxyisocaproate (fatty acid [FA] 6:0;OH), 3-hydroxyoctanoate (FA 8:0;3OH), hydroxyisobutyrate (FA 4:0;OH), C4-OH carnitine, p-hydroxyphenylacetate, phenyllactate, N-carbamoyl-beta-alanine, and N6-N6-N6-trimethyllysine. C4-OH-carnitine did not reach significance in the within-cohort validation ($p = 0.2$), likely due to the smaller number of deaths (64 deaths) compared to the Vietnamese external validation cohort (103 deaths). We additionally identified sebacate (a dicarboxylic acid) in a sensitivity analysis when using the Vietnamese cohort for discovery (screening $p < 0.001$ and within-cohort validation $p = 0.014$) and Indonesian cohort for external validation ($p < 0.001$). Notably, these associations persisted when analyzed for 6-month mortality outcome (Table S3). In line with this, metabolite set (pathway) enrichment analysis identified amino acid turnover (specifically tryptophan) and lipid metabolism as top pathways associated with day 60 mortality (Table S4). Overall, our analysis therefore yielded a final list of 10 CSF metabolites, all positively associated with increased day 60 mortality (Figure 1A). N6-N6-N6-trimethyllysine and p-hydroxyphenylacetate showed heterogeneity, with a stronger association with mortality in the Indonesian cohort than the Vietnam cohort. Clustering analysis revealed these metabolites as two distinct yet related clusters separate from tryptophan (Figure 1B). The first cluster included the short- and medium-chain hydroxylated FA 6:0;OH, FA 8:0;OH, and FA 4:0;3OH and CAR C4-OH (FA transporter), and the second cluster consisted of metabolites involved in amino acid metabolism and degradation metabolites (p-hydroxyphenylacetate, phenyllactate, N-carbamoyl-beta-alanine, N6,N6,N6-trimethyllysine) and sebacate (a dicarboxylic acid) (Table S5).

Comparison of metabolite concentrations between TBM, cryptococcal meningitis, bacterial meningitis, and non-infectious controls was limited by relatively small numbers in the non-TBM groups. However, CSF concentrations of metabolites were higher in those with TBM than in non-infectious controls, except for the hydroxylated FAs (Figure 1C).

Relative contribution of CSF metabolites to TBM mortality

We performed variable selection analysis to determine the relative importance of CSF metabolites and other clinical variables associated with TBM day 60 mortality. The analysis showed that pre-treatment disease severity (Glasgow Coma Scale [GCS]) had the strongest association with mortality, followed by the metabolites FA 8:0;3OH and tryptophan. These three variables were more strongly associated with death than the known risk factors HIV status and age (Figure 2A).

Patients with more severe TBM, as assessed by MRC grading, had higher CSF concentrations of all mortality-associated metabolites (Table S6). Variable selection result suggests, however, that the associations between CSF FA 8:0;3OH and tryptophan with mortality were independent from disease severity. In other words, among patients with similar GCS scores (i.e., with similar conscious level), those with higher levels of CSF FA 8:0;3OH or tryptophan had a greater risk of death (Figure 2B).

We further investigated whether TBM severity serves as an intermediate factor for the metabolite-mortality associations, as depicted in the causal diagram in Figure 2C, using mediation analysis. While variations in most pre-treatment metabolites directly impacted TBM mortality independent of TBM severity, five metabolites (C4-OH carnitine, tryptophan, p-hydroxyphenylacetate, phenyllactate, and N-carbamoyl-beta-alanine) had approximately 11%–23% of their variations affecting TBM mortality through TBM severity (Figure 2D), using MRC grading as a mediator (Table S7).

Relative abundance of CSF, serum FAs, and carnitines in TBM and non-infectious controls

CSF metabolites may be directly derived from the systemic circulation, especially with a damaged blood-brain barrier.[24] To explore the relationship between CSF and circulating (serum) metabolite levels in TBM and non-infectious controls, we reanalyzed data from a previous cohort[12] for the 10 metabolites associated with mortality. This showed that lower CSF tryptophan levels in TBM were accompanied by slightly higher serum tryptophan (Figure 3A). Three hydroxylated FAs (FA 4:0;OH, FA 6:0;OH, and FA 8:0;3OH) showed no clear differences in CSF, but their serum levels were lower in TBM patients compared to non-infectious controls. On the other hand, levels of CAR 4:0;OH were significantly higher in CSF but lower in serum when comparing TBM and non-infectious controls. Additionally, levels of CSF phenyllactate, N-carbamoyl-beta-alanine, and N6-N6-N6-trimethyllysine were higher in TBM compared to non-infectious controls, but we did not observe differences in serum values for these metabolites.

We extended the analysis of CSF and serum metabolite levels to the whole family of annotated FAs (including monocarboxylic [i.e., non-hydroxylated], dicarboxylic, and hydroxylated FAs) and

Table 1. Baseline clinical characteristics of study participants in Indonesia and Vietnam

Characteristics	n[a]	Indonesia (n = 388)	n[a]	Vietnam (n = 679)	p Value[b]
Female sex, no. (%)	388	156 (40%)	679	213 (31%)	0.004
Age, median (first, third interquartile), years	388	30 (23, 38)	679	36 (29, 47)	<0.001
Diagnostic category, no. (%)	388	–	679	–	0.033
Definite TBM	–	233 (60%)	–	452 (67%)	–
Probable TBM	–	155 (40%)	–	227 (33%)	–
HIV positive, no. (%)	388	34 (8.8%)	679	250 (37%)	<0.001
Modified MRC disease severity grade[c] — no. (%)	369	–	679	–	<0.001[d]
I	–	30 (8.1%)	–	257 (38%)	–
II	–	284 (76.9%)	–	298 (44%)	–
III	–	55 (15%)	–	124 (18%)	–
Median score on the GCS (first, third interquartile)	388	13 (12, 15)	679	15 (12, 15)	<0.001
CSF parameters					
Total leukocytes, cells/μL	388	161 (49, 344)	678	144 (48, 328)	0.6
Polymorphonuclear leukocyte counts, cells/μL	388	34 (8, 116)	653	15 (0, 88)	<0.001
Mononuclear cells, cells/μL	388	90 (33, 181)	653	103 (40, 217)	0.036
Protein level, g/L	388	178 (102, 328)	671	130 (80, 200)	<0.001
CSF to blood glucose ratio	388	0.20 (0.12, 0.32)	671	0.30 (0.20, 0.40)	<0.001
Mean GeneXpert Ct value	127	29.7 (26.1, 40.0)	654	40.0 (29.0, 40.0)	<0.001
Outcome					
Sixty-day mortality, no. of deaths/total no. of patients (%)	388	127/388 (33%)	679	103/679 (15%)	<0.001[e]

[a]Number of observations; median (first, third interquartile) for continuous variables and frequency (%) for categorical variables.
[b]Pearson's chi-square test for category variables; Wilcoxon rank-sum test for continuous variables.
[c]UK MRC scale on severity of TBM.[22]
[d]p value of Pearson's chi-square test for comparison between grade (I vs. II and III) and population.
[e]Log rank test for 2-month mortality outcome.

carnitines using present data and previous cohorts.[12] Concentrations of small- and medium-chain non-hydroxylated (monocarboxylic) FAs in TBM patients were higher in TBM compared to non-infectious controls, with the largest differences for FAs with a carbon chain length of 16 and above, including arachidonic acid (FA 20:4). Similarly, carnitines coupled to monocarboxylic FAs showed higher levels in TBM patients compared to controls. Monocarboxylic FAs and their carnitines however, showed limited correlation to outcome (Figure 3B).

Carnitines coupled to hydroxylated FAs, strongly correlated with CSF total protein and moderately with CSF bacterial load (Figures S2.1 and S2.2). The hydroxylated FAs themselves showed a unique pattern: no difference between TBM and non-infectious controls but much higher levels of hydroxylated FAs and their carnitines in non-survivors.

Correlation of top-hit metabolites with clinical and CSF inflammatory markers

As observed previously,[13] CSF tryptophan did not correlate strongly with other CSF or clinical parameters. Unlike tryptophan, the newly identified metabolites displayed strong associations with clinical severity (with higher levels in patients with a lower GCS score) and with CSF protein (a proxy marker for blood-brain barrier disruption[25]) (Figure 4). These metabolites showed weak or absent correlations with CSF mononuclear or polymorphonuclear cell counts and a weak positive correlation with CSF bacterial load and CSF inflammatory cytokines concentrations (tumor necrosis factor alpha, interleukin-2 [IL-2], IL-6, IL-4, IL-5, IL-13, and IL-10). We found lower concentrations of CSF FAs and C4-OH carnitine but higher concentrations of CSF tryptophan and N-carbamoyl-beta-alanine in HIV-positive than HIV-negative patients (Table S6).

DISCUSSION

We applied untargeted metabolome analysis of pre-treatment CSF samples from 1,069 Indonesian and Vietnamese adults with TBM to identify metabolites associated with mortality and gained insight into the underlying pathophysiology. Using a re-sampling approach and validation in two cohorts, we identified 10 metabolites robustly associated with mortality. Among these metabolites was tryptophan, which we had identified previously as being strongly associated with mortality.[12,13] The nine newly identified metabolites formed two clusters. The first cluster comprised FAs and a carnitine, with the hydroxylated FA 8:0;3OH showing the strongest association with mortality, independent of the known prognostic factors, including GCS score, HIV status, and tryptophan levels. The second cluster consisted of metabolites involved in amino acid metabolism and degradation pathways.

Concentrations of most non-hydroxylated (monocarboxylic) FAs were higher in CSF and lower in serum in TBM compared

Table 2. CSF metabolites associated with mortality in both cohorts

	Within-cohort validation[a] (Indonesia)				External validation (Vietnam)				Whole Indonesian cohort[a]			
Metabolites	HR[b]	95% CI[c]	p-value	FDR[c]	HR[b]	95% CI[b]	p-value	FDR	HR[b]	95% CI[b]	p-value	Test for heterogeneity[d]
FA 6:0; OH (hydroxyisocaproate)	1.47	1.15, 1.87	0.002	0.016	1.48	1.16, 1.88	0.002	0.028	1.60	1.35, 1.90	<0.001	0.594
FA 8:0; 3OH (3-hydroxyoctanoate)	1.36	1.06, 1.74	0.014	0.054	1.51	1.26, 1.81	<0.001	0.001	1.41	1.20, 1.66	<0.001	0.617
FA 4:0; OH (hydroxyisobutyrate)	1.35	1.04, 1.76	0.023	0.068	1.46	1.15, 1.86	0.002	0.033	1.53	1.25, 1.87	<0.001	0.754
CAR 4:0; OH (C4-OH carnitine)	1.21	0.91, 1.63	0.2	0.255	1.51	1.15, 1.99	0.003	0.046	1.46	1.17, 1.83	<0.001	0.902
Tryptophan	1.32	1.04, 1.67	0.021	0.064	1.42	1.17, 1.72	<0.001	0.009	1.37	1.16, 1.61	<0.001	0.838
p-hydroxyphenylacetate	1.46	1.12, 1.91	0.006	0.038	1.33	1.09, 1.61	0.004	0.049	1.67	1.42, 1.97	<0.001	0.042
Phenyllactate	1.62	1.23, 2.15	<0.001	0.01	1.55	1.24, 1.94	<0.001	0.007	1.77	1.45, 2.16	<0.001	0.302
N-carbamoyl-beta-alanine	1.56	1.25, 1.95	<0.001	0.003	1.45	1.19, 1.76	<0.001	0.007	1.49	1.29, 1.71	<0.001	0.665
N6-N6-N6-trimethyllysine	1.83	1.40, 2.39	<0.001	0.001	1.36	1.08, 1.72	0.009	0.091	1.84	1.56, 2.17	<0.001	0.022

CI, confidence interval; HR, hazard ratio; FDR, false discovery rate.
[a]The Indonesian cohort was split into a screening and a within-cohort validation set of equal sizes ($n = 194$), and the table represents the results from the within-cohort validation (first column) and whole Indonesian cohort (third column).
[b]HR per 1 SD increase in metabolite abundance (metabolites were log2 transformed and normalized to an SD of 1).
[c]FDR for the validation of 107 metabolites from the Indonesian discovery cohort.
[d]Test for study population (Indonesia and Vietnam) as effect modifier.

to non-infectious controls. The preferential upregulation of CSF non-hydroxylated FA concentrations (which were not associated with mortality) may represent a physiological adaptation to elevated energy demands in a low-glucose brain environment, as typically observed in TBM. For example, octanoate (FA 8:0; the non-hydroxylated form of FA 8:0;3OH) can stimulate astrocyte production of the beneficial neuronal energy substrates lactate and ketone bodies energy supply.[26] Transfer of FAs from the cytosol into the mitochondria (Box 1; Figure 5) is mediated by carnitines,[27,28] whose CSF concentrations were highly elevated in TBM compared to non-infectious controls. These FAs and their carnitines are most likely transported over the blood-brain barrier by specific transporters rather than across the blood-CSF barrier, which lacks these transporters.[24] Our CSF findings are therefore likely to reflect metabolism of the brain parenchyma rather than just the CSF compartment of these patients. Serum concentrations of non-hydroxylated (monocarboxylic) FAs were lower in TBM compared to non-infectious controls, which might be due to increased transport to the central nervous system, enhanced β-oxidation outside of the central nervous system, or reduced food intake of severely ill TBM patients.

The hydroxylated FAs, which are exclusively elevated in non-surviving TBM patients, may represent intermediate metabolites generated during the second step of β-oxidation, in which a hydroxyl group is added to the β carbon position 3.[29] Their accumulation in CSF might therefore indicate dysregulation in β-oxidation and mitochondrial integrity. Under high energy demand during immune cell activation in TBM, astrocyte or microglial β-oxidation (FA oxidation) and the resulting lactate and ketone body production may fall short in adequate energy supply to neurons.[28] Neurons themselves can also utilize β-oxidation, but the resulting increase in reactive oxygen species may lead to DNA damage and cell death, as shown in neurodegenerative diseases.[17] FAs, including hydroxylated FAs, show only a weak correlation with CSF total protein (a proxy for barrier integrity[25]), making passive leakage over a disintegrated blood-CSF barrier less likely. The hydroxylated FAs specifically are only increased in non-surviving TBM patients, which further points to dysregulated mitochondrial β-oxidation as a contribution mechanism in these patients.

Increased CSF metabolite concentrations might also be caused by production from *M. tuberculosis*. However, all of our identified CSF metabolites were also identified in the CSF of non-infectious controls, indicating that they are more likely to be host derived, and showed moderate or no correlation with *M. tuberculosis* load. The second cluster consisted of phenyllactate and p-hydroxyphenylacetate, involved in phenylalanine metabolism and associated with neurodegenerative diseases such as phenylketonuria[30]; N-carbamoyl-beta-alanine, which induces reactive oxygen species production in neuronal cell culture[31]; and N6-N6-N6-trimethyllysine, which may be relevant as an epigenetic marker of histone modification (the methylated form of lysine) or as a carnitine precursor.[32] All 10 metabolites, including tryptophan, showed very weak or no significant associations with CSF cell counts. This suggests that the CSF metabolite findings partially reflect CSF cell metabolism but predominantly represent brain metabolism, including that of its immune cells and astrocytes. Notably, the absolute number of astrocytes in the brain is estimated to be over 1,000 times greater than the number of leukocytes in the CSF. Interestingly, CSF tryptophan did not correlate with the other nine metabolites associated with mortality. Previously measured using a targeted approach,[13] we confirm the robust association between TBM

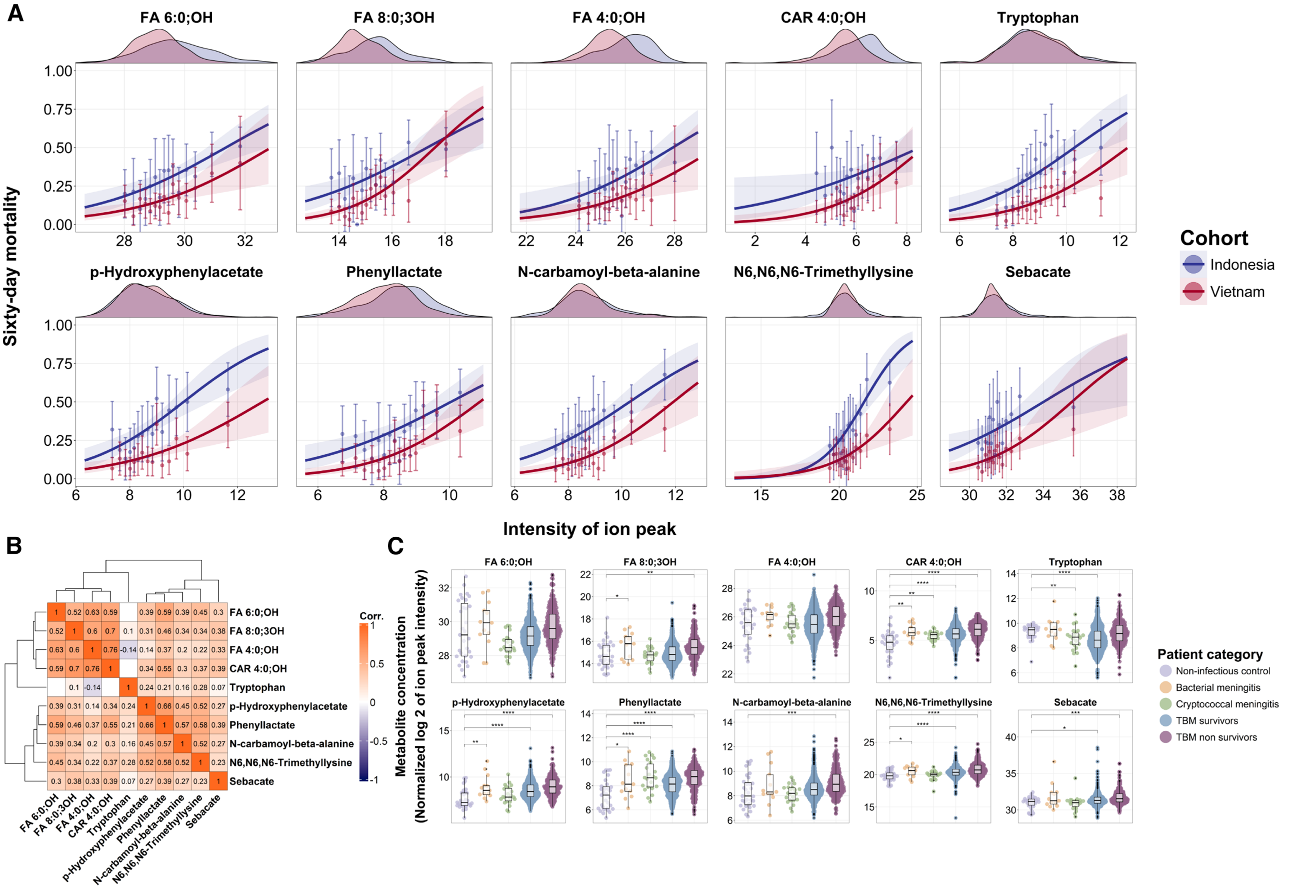

Figure 1. Pre-treatment metabolites and TBM 60-day mortality and comparison with other patient groups

(A) Relationship between metabolite abundance (normalized log2 transformed) and estimated mortality (logistic curve). For each metabolite, the distribution per cohort is visualized at the top of each panel and below the approximated association between that metabolite and estimated 60-day mortality. Metabolite levels were binned into 15 groups with equidistant quantile intervals (1/15, …, 14/15, 1). Points in each bin show the estimate of 2-month mortality per cohort with an error bar for the confidence interval of the estimate. 12 TBM patients lost to follow-up during the first 60 days were excluded from the plots.

(B) Correlation plot for the 10 identified metabolites with Spearman's correlations using hierarchical clustering. Insignificant correlations (Spearman $p > 0.05$) were left blank.

(C) Bee swarm boxplots show the distribution for the identified metabolites per patient group (non-infectious control, TBM, cryptococcus meningitis, bacterial meningitis, and TBM), stratified for 60-day mortality. 12 TBM patients lost to follow-up during the first 60 days were excluded from the plots. Distributions were compared based on Wilcoxon rank-sum test, with significance level denoted as $^{*}p \leq 0.05$, $^{**}p \leq 0.01$, $^{***}p \leq 0.001$, and $^{****}p \leq 0.0001$ without correction for multiple testing.

mortality and CSF tryptophan. While CSF tryptophan levels were lower in TBM patients compared to non-infectious controls, the newly identified metabolites (except FA 4:0;OH and FA 6:0;3OH) were higher in TBM patients. Taken together, these findings indicate that the newly identified metabolites exert their effects independent of the tryptophan pathway.

The strengths of our study include linking of untargeted liquid chromatography-mass spectrometry (LC-MS) CSF metabolomic profiles and detailed clinical characteristics and mortality data. These data, obtained from two different and large cohorts of adults with TBM, allowed for a robust discovery and validation approach. Using these cross-sectional pre-treatment metabolomics data, we leverage the heterogeneity of pre-treatment patient characteristics to develop hypotheses about potential pathways leading to death. Additionally, by incorporating known clinical predictors (GCS, HIV, age, and CSF bacterial burden) and a metabolic predictor (tryptophan) in our variable selection, we showed that CSF FA 8:0;3OH concentrations provide additional prognostic power. Mediation analysis showed that a small proportion of the variation of five metabolites (N-carbamoyl-beta-alanine, phenyllactate, p-hydroxyphenylacetate, tryptophan, and C4-OH carnitine) impacted mortality indirectly, mediated through TBM severity. This finding lays a foundation for further studies to identify the underlying mechanisms behind these associations, including experimental studies using animal models of TBM and brain injury. Additionally, our planned genetic mapping quantitative trait locus study for these 10 top-hit metabolites, along with colocalization analysis to determine whether the same genetic variants are associated with TBM mortality, may provide further insights.

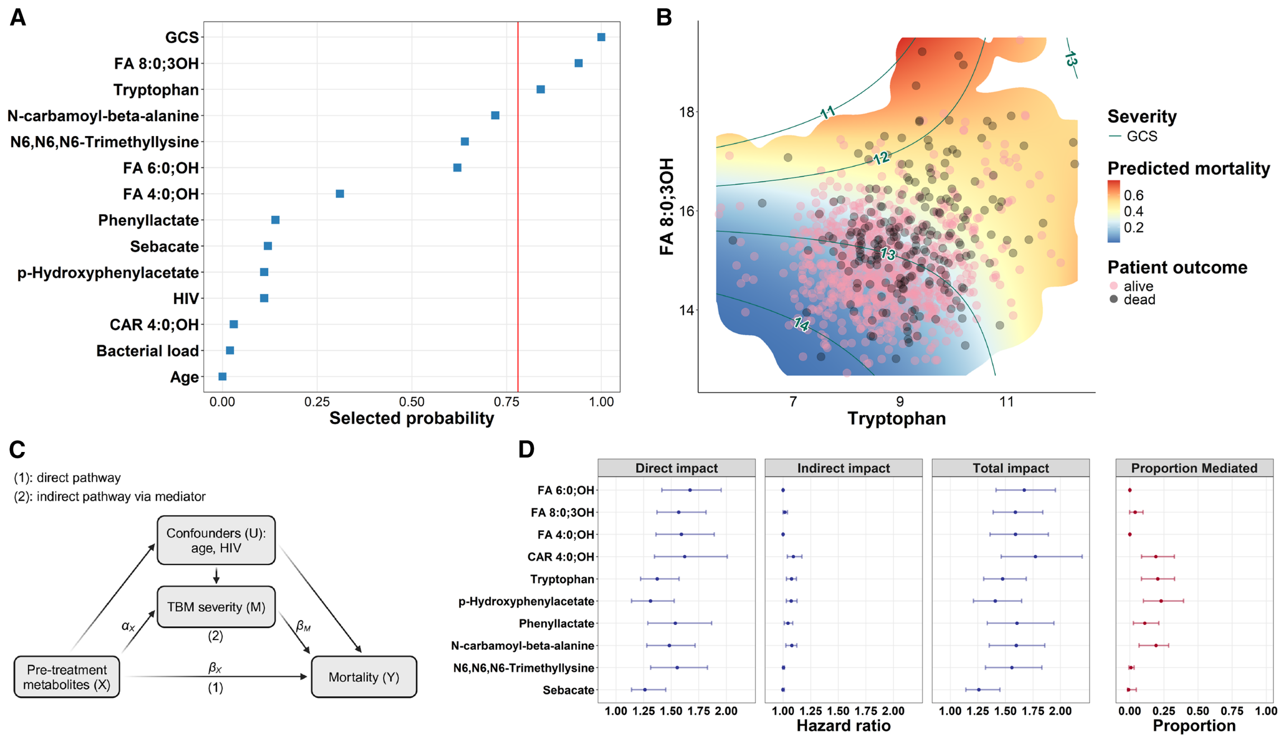

Figure 2. Prediction of CSF metabolites and other factors to TBM mortality

(A) Stability variable selection through multivariable analysis. The red line indicates a probability of 0.80 of the variable to be selected in the model, which is used to control the per-family error rate.[23] Bacterial load (GeneXpert Ct values) data were missing for 27% of patients. Variable selection analysis without bacterial load yielded the same result.

(B) The scatterplot visualizes TBM patients, following their log2-transformed CSF tryptophan (*x* axis) and FA 8:0;3OH (*y* axis) concentrations, colored according to their day 60 outcome (pink, alive; black, dead). The plot is superimposed onto a heatmap showing predicted 60-day mortality, modeled through logistic regression with the two metabolites and their interaction effect as covariates. The heatmap only visualizes the region with data points corresponding to the support from 2D kernel density estimates of the two metabolites. The green Glasgow Coma Scale (GCS) contour lines are based on a proportional odds logistic regression model, with the GCS score as the outcome and the two metabolites, along with their interaction effect, as covariates. Individuals within the same contour interval had similar levels of consciousness.

(C) Causal diagram showing the hypothesized relationship between CSF metabolites, TBM severity, and mortality.

(D) Forest plots visualizing the estimates of direct and indirect impact of all top-hit metabolites on mortality. Dots represent point estimates, and error bars indicate their confidence intervals. Shown on the far right is the proportion of the total impact that is mediated indirectly (i.e., through TBM disease severity).

Limitations of the study

Information on a patient's diet prior to sampling was not available. Another limitation is that the applied LC-MS cannot resolve all isomers; for instance, it does not resolve the ketone body β-hydroxybutyrate (FA 4:0;3OH) from and α-hydroxybutyrate (FA 4:0;2OH). The study would also have befitted from longitudinal CSF samples, as metabolites associated with the resolution of inflammation may also be relevant to understand pathogenesis.[33] Inclusion of follow-up CSF samples, which were unavailable in our cohorts, could help us to move beyond the associations with mortality we inferred from samples before the start of treatment.

In conclusion, we identified and validated nine new metabolites associated with TBM mortality in addition to tryptophan. We hypothesize that, in the typical low CSF glucose environment in patients with TBM, the increase in hydroxylated FAs and carnitines in those who die reflects a dysregulation in β-oxidation, causing secondary neurotoxicity. Future studies should examine whether interventions targeting cerebral metabolism or oxygenation can improve survival of TBM.

RESOURCE AVAILABILITY

Lead contact

Requests for further information and resources should be directed to and will be fulfilled by the lead contact, Nguyen Thuy Thuong Thuong (thuongntt@oucru.org).

Materials availability

This study did not generate new unique reagents.

Data and code availability

- The normalized peak ion intensities together with patient data (diagnostic group, age, sex, 60-day survival status, time-to-event, and HIV status) are available in Table S8 and at the NIH Common Fund's National Metabolomics Data Repository Website, the Metabolomics Workbench (https://www.metabolomicsworkbench.org), where it has

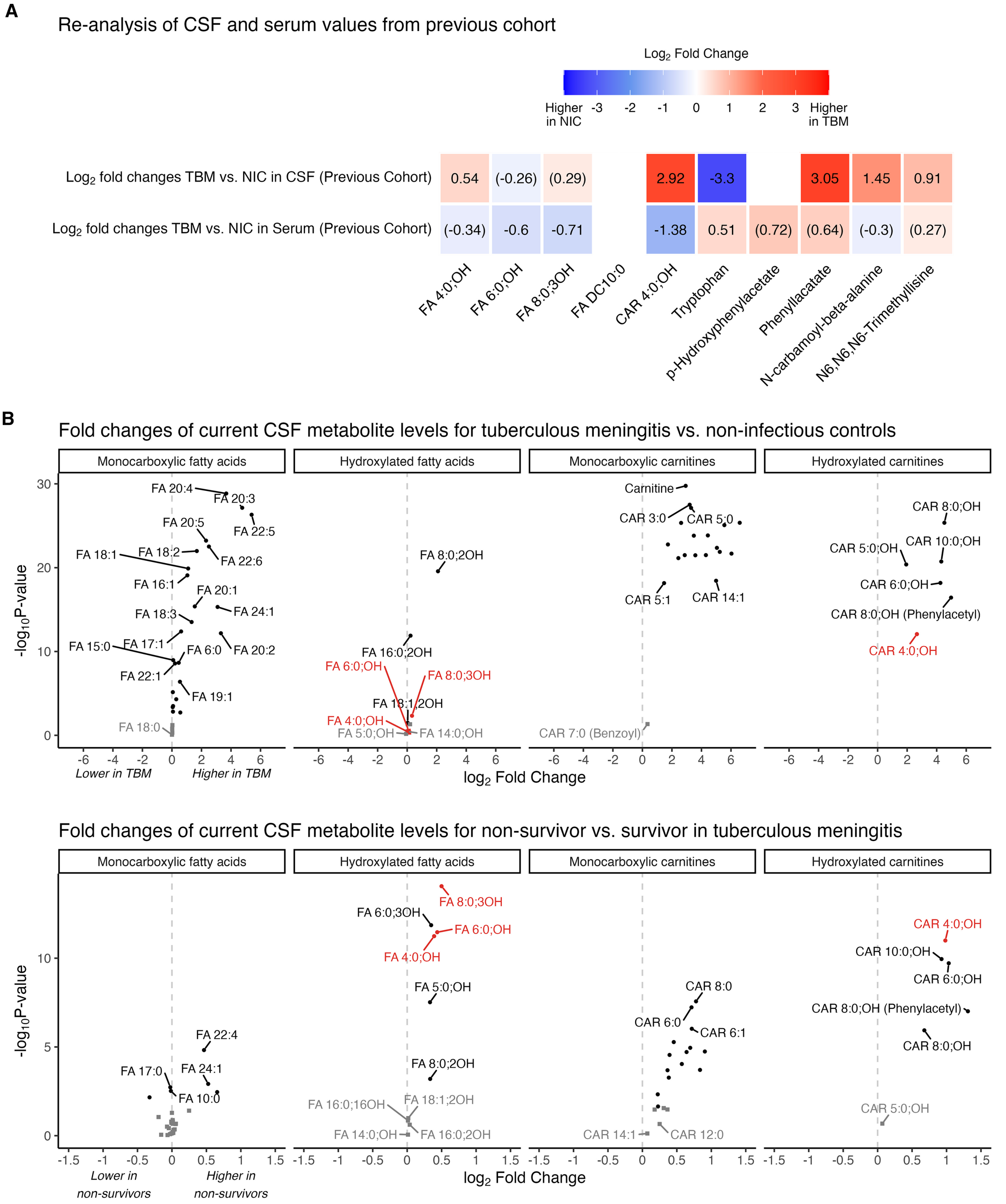

Figure 3. Re-analysis of CSF and serum values from a previous cohort and fold changes of current CSF FAs and carnitines

(A) Log2 fold changes for TBM patients vs. non-infectious controls (NICs) in CSF and serum analyses in a previous cohort.[12] Fold changes with unadjusted $p > 0.05$ are enclosed in parentheses. Fold change for sebacate (FA DC10:0) and p-hydroxyphenylacetate could not be calculated, as >25% of measurements were below the detection limit in the TBM or NIC patient groups.

(legend continued on next page)

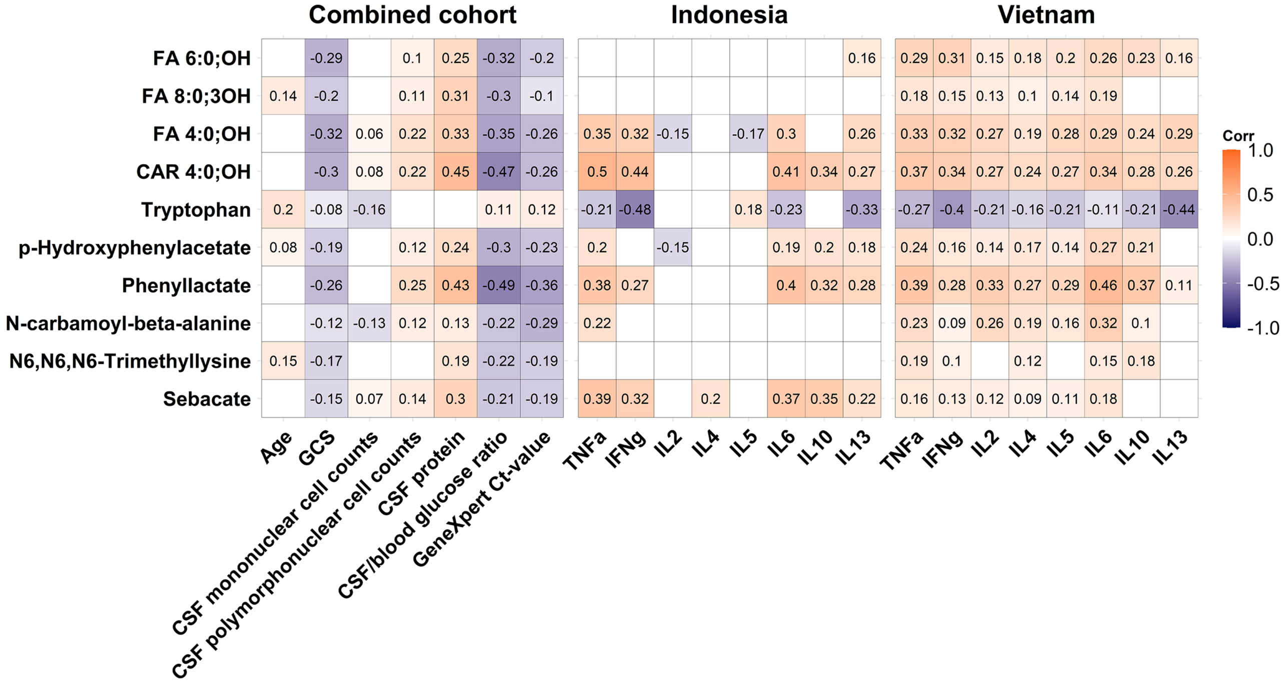

Figure 4. Metabolite clustering and correlation with clinical characteristics
Shown on the left are Spearman's correlations between identified metabolites and clinical characteristics, including age, GCS score, CSF cell counts, CSF protein (as a proxy for barrier breakdown), CSF/blood glucose ratio, and GeneXpert Ct value (bacterial load). Significant correlations ($p < 0.05$) were visualized in a heatmap, while insignificant correlations were left blank. The order of metabolites was based on hierarchical clustering by computing the Spearman's correlation coefficient between metabolites. Shown in the center and on the right are Spearman's correlations between top-hit metabolites and log2-transformed CSF cytokine levels, measured using a proximity extension assay (O-link) in 257 Indonesian TBM patients and using Luminex in 756 Vietnamese TBM patients, respectively.

been assigned project ID P002365 and study ID ST003788. The data can be accessed directly (https://doi.org/10.21228/M8XN97).

- All original code has been deposited at Zenodo at https://doi.org/10.5281/zenodo.14880570 and is publicly available as of the date of publication.
- Any additional information required to reanalyze the data reported in this paper is available from the lead contact upon request.

ACKNOWLEDGMENTS

The authors thank the neurology residents and Tiara Pramaesya, Sofia Immaculata, Putri Andini, Sri Margi, Rani Trisnawati, and Shehika Shulda of the tuberculous meningitis study team for monitoring patients and data management; Lidya Chaidir and Jessi Annisa for mycobacterial diagnostics; and the director of the Hasan Sadikin General Hospital, Bandung, Indonesia, for accommodating the research. The authors thank Prof. Jelle Goeman for statistical advice on the ranking method. This study was supported by the National Institutes of Health (R01AI145781) and the Wellcome Trust (110179/Z/15/Z and 206724/Z/17/Z). Previous establishment of the cohorts in Indonesia was supported by the Direktorat Jendral Pendidikan Tinggi (BPPLN fellowship to S.D.); the Ministry of Research, Technology, and Higher Education, Indonesia (PKSLN grant to R.R. and S.D.); and the United States Agency for International Development (PEER Health grant to R.R.). The Metabolomics Data Repository is supported by the Metabolomics Workbench/National Metabolomics Data Repository (NMDR) (grant U2C-DK119886), the Common Fund Data Ecosystem (CFDE) (grant 3OT2OD030544), and the Metabolomics Consortium Coordinating Center (M3C) (grant 1U2C-DK119889). The funders had no role in study design, data collection and analysis, decision to publish, or preparation of the manuscript.

AUTHOR CONTRIBUTIONS

Conceptualization, L.T.H.N., J.A.-P., R.v.C., G.T., C.B.C., N.T.T.T., and A.v.L.; methodology, J.A.-P. and C.B.C.; formal analysis, E.A., K.C.J.v.A., E.A., G.C., H.T.H., N.T.B.M., and T.M.T.; investigation, A.D., J.K., J.P., L.D., and T.T.B.T.; writing – original draft, L.T.H.N., K.C.J.v.A., J.A.-P., N.T.T.T., and A.v.L.; writing – review & editing, L.T.H.N., K.C.J.v.A., J.A.-P., L.S., M.G.N., V.K., R.v.C., G.T., C.B.C., N.T.T.T., and A.v.L.; funding acquisition, J.A.-P., R.v.C., G.T., C.B.C., V.K., R.L.H., N.T.T.T., and A.v.L.; resources, B.A., R.E., J.D., D.H., T.T.H.C., N.D.B., A.R.G., R.L.H., R.R., D.I., and K.M.; supervision, N.T.T.T. and A.v.L. N.T.T.T. and A.v.L. had unrestricted access to all data. All authors agreed to submit the manuscript, read and approved the final draft, and take full responsibility for its content, including the accuracy of the data.

DECLARATION OF INTERESTS

The authors declare no competing interests.

(B) Fold change of CSF metabolite levels in the current dataset for TBM patients versus NICs (top) and for TBM survivors versus non-survivors (bottom) for the different classes of FAs (left) and FAs coupled to their carnitines (right). Metabolites with adjusted $p < 0.05$ are shown in black circles and others in dark-gray squares, while those found from the primary analysis are highlighted in red.
See also Figure S2 and Table S8.

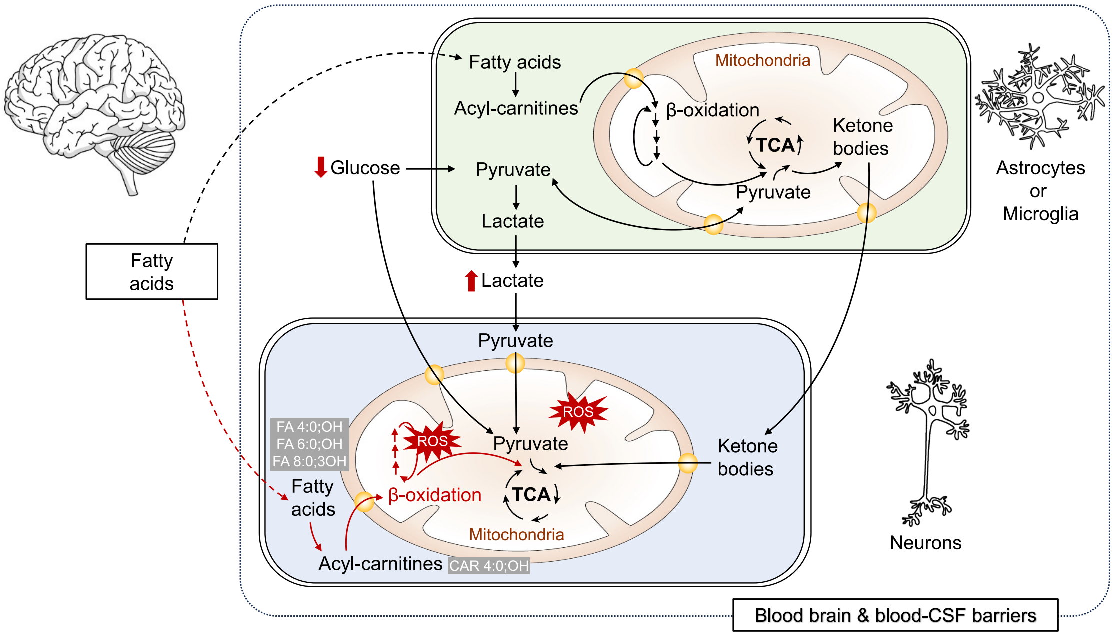

Figure 5. Dysregulated neuronal β-oxidation associated with TBM mortality

The diagram shows dysregulated neuronal β-oxidation in astrocytes, microglia, or neurons as a potential source of the hydroxylated FAs that were associated with increased TBM mortality. FAs may be used by astrocytes or microglia as an energy source in a low-glucose environment, from which ketone bodies may be generated to support neurons. However, this support may be insufficient, leading to the use of β-oxidation and the accumulation of reactive oxygen species (ROS) in neurons, damaging the cells. The diagram highlights the hydroxylated FAs (FA 4:0;OH, FA 6:0;OH, and FA 8:0;3OH) and carnitine (CAR 4:0;OH) identified to predict mortality. FAs are transported into the mitochondria as acyl-carnitines. Hypothesized negative effects in neurons are highlighted in red.

STAR★METHODS

Detailed methods are provided in the online version of this paper and include the following:

- KEY RESOURCES TABLE
- EXPERIMENTAL MODEL AND STUDY PARTICIPANT DETAILS
  - Study population
- METHOD DETAILS
  - Sample processing and liquid chromatography-mass spectrometry
- QUANTIFICATION AND STATISTICAL ANALYSIS
  - A two-cohort validation strategy
  - Metabolite data quality control and data preprocessing
  - Descriptive analysis
  - Survival analysis and resampling ranking procedure
  - Pathway analysis
  - Clustering and association analysis of metabolites with clinical characteristics
  - Comparison of metabolites abundance with other groups of meningitis patients
  - Variable selection analysis - Multivariate analysis
  - Mediation analysis
  - Re-analysis of serum and CSF levels of top-hit metabolites for fatty acids and carnitines

SUPPLEMENTAL INFORMATION

Supplemental information can be found online at https://doi.org/10.1016/j.medj.2025.100692.

Received: July 25, 2024
Revised: January 4, 2025
Accepted: April 24, 2025
Published: May 23, 2025

REFERENCES

1. Stadelman, A.M., Ellis, J., Samuels, T.H.A., Mutengesa, E., Dobbin, J., Ssebambulidde, K., Rutakingirwa, M.K., Tugume, L., Boulware, D.R., Grint, D., and Cresswell, F.V. (2020). Treatment outcomes in adult tuberculous meningitis: A systematic review and meta-analysis. Open Forum Infect. Dis. *7*, ofaa257.
2. Dodd, P.J., Osman, M., Cresswell, F.V., Stadelman, A.M., Lan, N.H., Thuong, N.T.T., Muzyamba, M., Glaser, L., Dlamini, S.S., and Seddon, J. A. (2021). The global burden of tuberculous meningitis in adults: A modelling study. PLOS Glob. Public Health *1*, e0000069.
3. Huynh, J., Donovan, J., Phu, N.H., Nghia, H.D.T., Thuong, N.T.T., and Thwaites, G.E. (2022). Tuberculous meningitis: progress and remaining questions. Lancet Neurol. *21*, 450–464.
4. Thuong, N.T.T., Vinh, D.N., Hai, H.T., Thu, D.D.A., Nhat, L.T.H., Heemskerk, D., Bang, N.D., Caws, M., Mai, N.T.H., and Thwaites, G.E. (2019). Pretreatment cerebrospinal fluid bacterial load correlates with inflammatory response and predicts neurological events during tuberculous meningitis treatment. J. Infect. Dis. *219*, 986–995.
5. Rohlwink, U.K., Mauff, K., Wilkinson, K.A., Enslin, N., Wegoye, E., Wilkinson, R.J., and Figaji, A.A. (2017). Biomarkers of cerebral injury and inflammation in pediatric tuberculous meningitis. Clin. Infect. Dis. *65*, 1298–1307.

6. Van Laarhoven, A., Dian, S., Ruesen, C., Hayati, E., Damen, M.S.M.A., Annisa, J., Chaidir, L., Ruslami, R., Achmad, T.H., Netea, M.G., et al. (2017). Clinical parameters, routine inflammatory markers, and LTA4H genotype as predictors of mortality among 608 patients with tuberculous meningitis in Indonesia. J. Infect. Dis. *215*, 1029–1039.
7. Thwaites, G.E., Nguyen, D.B., Nguyen, H.D., Hoang, T.Q., Do, T.T.O., Nguyen, T.C.T., Nguyen, Q.H., Nguyen, T.T., Nguyen, N.H., Nguyen, T. N.L., et al. (2004). Dexamethasone for the Treatment of Tuberculous Meningitis in Adolescents and Adults. N. Engl. J. Med. *351*, 1741–1751.
8. Donovan, J., Bang, N.D., Imran, D., Nghia, H.D.T., Burhan, E., Huong, D.T. T., Hiep, N.T.T., Ngoc, L.H.B., Thanh, D.V., Thanh, N.T., et al. (2023). Adjunctive Dexamethasone for Tuberculous Meningitis in HIV-Positive Adults. N. Engl. J. Med. *389*, 1357–1367.
9. Mai, N.T.H., Dobbs, N., Phu, N.H., Colas, R.A., Thao, L.T.P., Thuong, N.T. T., Nghia, H.D.T., Hanh, N.H.H., Hang, N.T., Heemskerk, A.D., et al. (2018). A randomised double blind placebo controlled phase 2 trial of adjunctive aspirin for tuberculous meningitis in HIV-uninfected adults. Elife *7*, e33478.
10. Colas, R.A., Nhat, L.T.H., Thuong, N.T.T., Gómez, E.A., Ly, L., Thanh, H.H., Mai, N.T.H., Phu, N.H., Thwaites, G.E., and Dalli, J. (2019). Proresolving mediator profiles in cerebrospinal fluid are linked with disease severity and outcome in adults with tuberculous meningitis. FASEB J. *33*, 13028–13039.
11. Kumar, R., Singh, P., Kolloli, A., Shi, L., Bushkin, Y., Tyagi, S., and Subbian, S. (2019). Immunometabolism of Phagocytes During Mycobacterium tuberculosis Infection. Front. Mol. Biosci. *6*, 1–20.
12. van Laarhoven, A., Dian, S., Aguirre-Gamboa, R., Avila-Pacheco, J., Ricaño-Ponce, I., Ruesen, C., Annisa, J., Koeken, V.A.C.M., Chaidir, L., Li, Y., et al. (2018). Cerebral tryptophan metabolism and outcome of tuberculous meningitis: an observational cohort study. Lancet Infect. Dis. *18*, 526–535.
13. Ardiansyah, E., Avila-Pacheco, J., Nhat, L.T.H., Dian, S., Vinh, D.N., Hai, H. T., Bullock, K., Alisjahbana, B., Netea, M.G., Estiasari, R., et al. (2023). Tryptophan metabolism determines outcome in tuberculous meningitis: a targeted metabolomic analysis. Elife *12*, e85307–e85317.
14. Szrok-Jurga, S., Turyn, J., Hebanowska, A., Swierczynski, J., Czumaj, A., Sledzinski, T., and Stelmanska, E. (2023). The Role of Acyl-CoA β--Oxidation in Brain Metabolism and Neurodegenerative Diseases. Int. J. Mol. Sci. *24*, 13977.
15. Ebert, D., Haller, R.G., and Walton, M.E. (2003). Energy contribution of octanoate to intact rat brain metabolism measured by 13C nuclear magnetic resonance spectroscopy. J. Neurosci. *23*, 5928–5935.
16. Schönfeld, P., and Reiser, G. (2021). How the brain fights fatty acids' toxicity. Neurochem. Int. *148*, 105050.
17. Toomey, C.E., Heywood, W.E., Evans, J.R., Lachica, J., Pressey, S.N., Foti, S.C., Al Shahrani, M., D'Sa, K., Hargreaves, I.P., Heales, S., et al. (2022). Mitochondrial dysfunction is a key pathological driver of early stage Parkinson's. Acta Neuropathol. Commun. *10*, 134.
18. Tracey, T.J., Steyn, F.J., Wolvetang, E.J., and Ngo, S.T. (2018). Neuronal Lipid Metabolism: Multiple Pathways Driving Functional Outcomes in Health and Disease. Front. Mol. Neurosci. *11*, 10.
19. Farmer, B.C., Kluemper, J., and Johnson, L.A. (2019). Apolipoprotein E4 Alters Astrocyte Fatty Acid Metabolism and Lipid Droplet Formation. Cells *8*, 182.
20. Auestad, N., Korsak, R.A., Morrow, J.W., and Edmond, J. (1991). Fatty Acid Oxidation and Ketogenesis by Astrocytes in Primary Culture. J. Neurochem. *56*, 1376–1386.
21. Bixel, M.G., and Hamprecht, B. (1995). Generation of Ketone Bodies from Leucine by Cultured Astroglial Cells. J. Neurochem. *65*, 2450–2461.
22. Thwaites, G.E., van Toorn, R., and Schoeman, J. (2013). Tuberculous meningitis: More questions, still too few answers. Lancet Neurol. *12*, 999–1010. https://doi.org/10.1016/S1474-4422(13)70168-6.
23. Meinshausen, N., and Bühlmann, P. (2010). Stability selection. J. R. Stat. Soc. Ser. B Stat. Methodol. *72*, 417–473.
24. Carstens, G., Verbeek, M.M., Rohlwink, U.K., Figaji, A.A., Te Brake, L., and van Laarhoven, A. (2024). Metabolite transport across central nervous system barriers. J. Cereb. Blood Flow Metab. *44*, 1063–1077. https://doi.org/10.1177/0271678X241241908.
25. Svensson, E.M., Dian, S., Te Brake, L., Ganiem, A.R., Yunivita, V., van Laarhoven, A., Van Crevel, R., Ruslami, R., and Aarnoutse, R.E. (2020). Model-Based Meta-analysis of Rifampicin Exposure and Mortality in Indonesian Tuberculous Meningitis Trials. Clin. Infect. Dis. *71*, 1817–1823.
26. Thevenet, J., De Marchi, U., Domingo, J.S., Christinat, N., Bultot, L., Lefebvre, G., Sakamoto, K., Descombes, P., Masoodi, M., and Wiederkehr, A. (2016). Medium-chain fatty acids inhibit mitochondrial metabolism in astrocytes promoting astrocyte-neuron lactate and ketone body shuttle systems. FASEB J. *30*, 1913–1926.
27. Liu, K.H., Owens, J.A., Saeedi, B., Cohen, C.E., Bellissimo, M.P., Naudin, C., Darby, T., Druzak, S., Maner-Smith, K., Orr, M., et al. (2021). Microbial metabolite delta-valerobetaine is a diet-dependent obesogen. Nat. Metab. *3*, 1694–1705.
28. Loppi, S.H., Tavera-Garcia, M.A., Becktel, D.A., Maiyo, B.K., Johnson, K. E., Nguyen, T.V.V., Schnellmann, R.G., and Doyle, K.P. (2023). Increased fatty acid metabolism and decreased glycolysis are hallmarks of metabolic reprogramming within microglia in degenerating white matter during recovery from experimental stroke. J. Cereb. Blood Flow Metab. *43*, 1099–1114.
29. Talley, J.T., and Mohiuddin, S.S. (2024). Biochemistry, Fatty Acid Oxidation. In StatPearls [Internet] (Treasure Island (FL): StatPearls Publishing). https://www.ncbi.nlm.nih.gov/books/NBK556002/.
30. Blau, N., Van Spronsen, F.J., and Levy, H.L. (2010). Phenylketonuria. Lancet *376*, 1417–1427.
31. Kölker, S., Okun, J.G., Hörster, F., Assmann, B., Ahlemeyer, B., Kohlmüller, D., Exner-Camps, S., Mayatepek, E., Krieglstein, J., and Hoffmann, G. F. (2001). 3-Ureidopropionate contributes to the neuropathology of 3-ureidopropionase deficiency and severe propionic aciduria: A Hypothesis. J. Neurosci. Res. *66*, 666–673.
32. Maas, M.N., Hintzen, J.C.J., Porzberg, M.R.B., and Mecinović, J. (2020). Trimethyllysine: From Carnitine Biosynthesis to Epigenetics. Int. J. Mol. Sci. *21*, 9451.
33. Tomalka, J., Sharma, A., Smith, A.G.C., Avaliani, T., Gujabidze, M., Bakuradze, T., Sabanadze, S., Jones, D.P., Avaliani, Z., Kipiani, M., et al. (2024). Combined cerebrospinal fluid metabolomic and cytokine profiling in tuberculosis meningitis reveals robust and prolonged changes in immunometabolic networks. Tuberculosis *144*, 102462.
34. Therneau, T. (2021). A Package for Survival Analysis in R; Version 3.2-11. https://CRAN.R-project.org/package=survival.
35. Hofner B., and Hothorn T. (2021). stabs: Stability Selection with Error Control. R Package version 0.6-4. https://doi.org/10.1186/s1285901505753.See.
36. Sjoberg, D., Whiting, K., Curry, M., Lavery, J., and Larmarange, J. (2021). Reproducible Summary Tables with the gtsummary Package. R J. *13*, 570–580.
37. Wickham, H. (2010). ggplot2: Elegant Graphics for Data Analysis, *35* (New York: Springer-Verlag).
38. Shi, B., Choirat, C., Coull, B.A., Vanderweele, T.J., and Valeri, L. (2021). CMAverse: A Suite of Functions for Reproducible Causal Mediation Analyses. Epidemiology *32*, E20–E22, at. https://doi.org/10.1097/EDE.0000000000001378.
39. Wickham, H., Averick, M., Bryan, J., Chang, W., McGowan, L., François, R., Grolemund, G., Hayes, A., Henry, L., Hester, J., et al. (2019). Welcome to the {tidyverse. J. Open Source Softw. *4*, 1686.
40. Hothorn, T., Hornik, K., van de Wiel, M.A., and Zeileis, A. (2006). A {L}ego system for conditional inference. Am. Stat. *60*, 257–263.
41. Kassambara, A. (2023). rstatix: Pipe-Friendly Framework for Basic Statistical Tests. at https://rpkgs.datanovia.com/rstatix/.
42. Pang, Z., Lu, Y., Zhou, G., Hui, F., Xu, L., Viau, C., Spigelman, A.F., MacDonald, P.E., Wishart, D.S., Li, S., and Xia, J. (2024). MetaboAnalyst 6.0:

towards a unified platform for metabolomics data processing, analysis and interpretation. Nucleic Acids Res. *52*, W398–W406.

43. Heemskerk, A.D., Bang, N.D., Mai, N.T.H., Chau, T.T.H., Phu, N.H., Loc, P. P., Chau, N.V.V., Hien, T.T., Dung, N.H., Lan, N.T.N., et al. (2016). Intensified Antituberculosis Therapy in Adults with Tuberculous Meningitis. N. Engl. J. Med. *374*, 124–134.
44. Rohlwink, U.K., Chow, F.C., Wasserman, S., Dian, S., Lai, R.P., Chaidir, L., Hamers, R.L., Wilkinson, R.J., Boulware, D.R., Cresswell, F.V., et al. (2019). Standardized approaches for clinical sampling and endpoint ascertainment in tuberculous meningitis studies. Wellcome Open Res. *4*, 204.
45. Boyles, T., Stadelman, A., Ellis, J.P., Cresswell, F.V., Lutje, V., Wasserman, S., Tiffin, N., and Wilkinson, R. (2021). The diagnosis of tuberculous meningitis in adults and adolescents: protocol for a systematic review and individual patient data meta-analysis to inform a multivariable prediction model [version 3; peer review: 2 approved]. Wellcome Open Res *4*, 19.
46. Benjamini, Y., and Hochberg, Y. (1995). Controlling the False Discovery Rate: A Practical and Powerful Approach to Multiple Testing. J. R. Stat. Soc. B *57*, 289–300.
47. Harrell, F. E. J. & Slaughter, J. C. Biostatistics for Biomedical Research. Power 1–158 (2015).
48. Fan, J., and Lv, J. (2008). Sure independence screening for ultrahigh dimensional feature space. J. R. Stat. Soc. Ser. B Stat. Methodol. *70*, 849–911.
49. Wen, C., Pan, W., Huang, M., and Wang, X. (2018). Sure independence screening adjusted for confounding covariates with ultrahigh dimensional data. Stat. Sin. *28*, 293–317.
50. Saldana, D.F., and Feng, Y.S.I.S. (2018). An R package for sure independence screening in ultrahigh-dimensional statistical models. J. Stat. Softw. *83*.
51. Wilkinson, L. (2018). Visualizing Big Data Outliers Through Distributed Aggregation. IEEE Trans. Vis. Comput. Graph. *24*, 256–266.
52. Robins, J.M. (1987). New Approach To Causal Inference in Mortality Studies With a Sustained Exposure Period - Application To Control of the Healthy Worker Survivor Effect. Comput. Math. Appl. *14*, 923–945.

STAR★METHODS

KEY RESOURCES TABLE

REAGENT or RESOURCE	SOURCE	IDENTIFIER
Deposited data		
Normalized Ion peak intensity	This paper	Metabolomics Workbench Project ID PR002365
Software and algorithms		
TraceFinder v 3.3	Thermo Scientific	https://www.thermofisher.com/order/catalog/product/OPTON-31006?SID=srch-srp-OPTON-31006
Progenesis QI	Nonlinear Dynamics	https://www.nonlinear.com/progenesis/qi/
R 4.2.0/4.4.0	R Core Team (2024)	https://www.R-project.org/
survival	Therneau[34]	https://cran.r-project.org/web/packages/survival/
stabs	Hofner B[35]	https://cran.r-project.org/web/packages/stabs/
gtsummary	Sjoberg et al.[36]	https://www.danieldsjoberg.com/gtsummary/
ggplot2 (v. 3.5.1)	Wickham[37]	https://ggplot2.tidyverse.org
CMAverse	Shi et al.[38]	https://github.com/BS1125/CMAverse
tidyverse (v. 2.0.0)	Wickham et al.[39]	https://cran.r-project.org/web/packages/tidyverse/
coin (v.1.4–3)	Hothorn et al.[40]	https://cran.r-project.org/web/packages/coin/
rstatix (v. 0.7.2)	Kassambara[41]	https://cran.r-project.org/web/packages/rstatix/
MetaboAnalyst (v. 6.0)	Pang et al.[42]	https://www.metaboanalyst.ca/MetaboAnalyst/

EXPERIMENTAL MODEL AND STUDY PARTICIPANT DETAILS

Study population

We included patients with definite TBM (i.e., those with either microbial confirmation by Ziehl-Neelsen staining, positive CSF culture or GeneXpert) and probable TBM (i.e., those without microbial confirmation but fulfilling at least 2 out of the 3 criteria: CSF leukocytes $\geq$5 cells/μL, CSF/blood glucose ratio <0.5, and CSF protein >0.45 g/L). The discovery cohort was from Indonesia, including all patients from an observational study conducted at Hasan Sadikin hospital between 2007-2019.[13] The external validation cohort from Vietnam included all patients from a randomised clinical trial on intensified anti-tuberculosis regimens, who were treated at Hospital for Tropical Diseases and Pham Ngoc Thach Hospital in Ho Chi Minh City between 2011-2014.[43] All patients from both cohorts received anti-tuberculosis and corticosteroids treatment. Patients whose pre-treatment specimens were not available were excluded. The primary outcome for the current study was 60-day mortality, when most deaths attributable to TBM occur. Patients were followed up clinically or by phone calls for at least 180 days (6 months). We also included non-infectious control patients from the same clinical sites. Non-infectious controls from Indonesia had undergone lumbar punctures for suspected infections or subarachnoid bleeding, with routine clinical tests excluding an infectious cause. In Vietnam, non-infectious controls had an alternative diagnosis confirmed by lumbar puncture. None of the controls received anti-tuberculosis treatment. The HIV-negative patients with microbiologically confirmed bacterial meningitis and HIV-positive patients with cryptococcal meningitis patients were included from the same sites.

Ethical approval was obtained separately from the Ethical Committee of Hasan Sadikin Hospital, Faculty of Medicine, Universitas Padjadjaran, Bandung for the Indonesia cohort, and from the Oxford Tropical Research Ethics Committee in the United Kingdom and the Institutional Review Boards of the Hospital for Tropical Diseases and Pham Ngoc Thach Hospital for the Vietnam cohort. Written consent from Vietnamese patients, and oral consent from Indonesian patients were obtained from the original studies for storage of surplus sample, and for generating follow-up data. This consent was obtained from patients or close relatives of patients who were unconscious.

METHOD DETAILS

Sample processing and liquid chromatography-mass spectrometry

CSF samples were processed according to in-house developed protocols and according to the “Standardized approaches for clinical sampling and endpoint ascertainment in tuberculous meningitis studies”,[44] with the exception that centrifugation speed has changed over time, ranging from 865-3000 $\times$ g, for 15 min. The resulting supernatants were stored at -80°C. Metabolites in CSF

were measured using an untargeted liquid chromatography-tandem mass spectrometry (LC-MS) method, utilizing Nexera X2 U-HPLC systems (Shimadzu Scientific Instruments) coupled to Q Exactive/Exactive Plus orbitrap mass spectrometers (Thermo Fisher Scientific) (Figure S4). Metabolites were extracted from 10 μL of CSF using 90 μL of acetonitrile/methanol/formic acid (74.9:24.9:0.2 v/v/v) containing stable isotope-labeled internal standards (valine-d8, Sigma-Aldrich, St. Louis, MO; and phenylalanine-d8, Cambridge Isotope Laboratories, Andover, MA). The resulting samples were centrifuged (10 min, 9,000 × g, 4°C), and the supernatants were then directly injected onto a 150 × 2 mm, 3 μm Atlantis HILIC column 130 (Waters; Milford, MA). The column was eluted isocratically at a flow rate of 250 μL/min with 5% mobile phase A (10 mM ammonium formate and 0.1% formic acid in water) for 0.5 min, followed by a linear gradient to 40% mobile phase B (acetonitrile with 0.1% formic acid) over 10 min. To correct for mass spectrometry sensitivity drift and for quality control analyses, pairs of pooled samples generated using aliquots from all samples in the study were included every 20 samples. Metabolite abundance is quantified as relative concentration based on peak ion intensity. Because the metabolome consists of molecules with very different physical properties, we use four platforms, each with its own validated series of procedures using at least one dedicated instrument in the lab. Briefly, the methods include.

(1) **C8-pos.** Lipids and nonpolar metabolites extracted from 10 μL plasma or CSF using 190 μL of isopropanol containing an internal standard, separated using reversed phase C8 ultrahigh performance chromatography (U-HPLC), and analyzed using high resolution, full scan MS in the Measures >200 lipids of known identity and thousands of unknown peaks.
(2) **C18-neg.** Free fatty acids, bile acids, and metabolites of intermediate polarity extracted from 30 μL plasma or CSF using 90 μL of methanol containing an internal standard, separated using reversed phase C18 UHPLC, and analyzed using high resolution, full scan MS in the negative ion mode. Measures >100 metabolites of confirmed identity and thousands of unknown peaks.
(3) **HILIC-pos.** Amino acids, amino acid metabolites, acylcarnitines, dipeptides, and other cationic polar metabolites extracted from 10 μL plasma or CSF using 90 μL of 25% methanol/75% acetonitrile (MeOH/ACN) containing two internal standards, separated using hydrophilic interaction liquid chromatography (HILIC), and analyzed using high resolution, full scan MS in the positive ion mode. Measures >200 metabolites of known identity and thousands of unknown peaks.
(4) **HILIC-neg.** Sugars, organic acids, purines, pyrimidines, and other anionic polar metabolites extracted from 30 μL plasma or CSF using 120 μL of methanol containing internal standards, separated using HILIC) under basic conditions, and analyzed using high resolution, full scan MS in the negative ion mode. Measures >80 metabolites of known identity and thousands of unknown peaks.

Metabolomics data processing

Data acquired using HRAM systems will be processed using TraceFinder (v 3.3, Thermo Scientific) for supervised extraction of data on known metabolites and using Progenesis QI software (Nonlinear Dynamics) to detect unknown peaks, perform chromatographic retention time alignment, and integrate peak areas. Compound identities were determined by matching the m/z and retention time (RT) indices of unknown features to an in-house library containing m/z and RTs as well as MS/MS spectra for over two thousand reference standards. The retention time deviations between the reference library and the unknown metabolites in CSF were estimated using in-house feature alignment algorithms as well as directly matching the RTs of unknowns to compounds in reference standards mixtures routinely measured in human biofluids for each profiling method. For certain molecules for which commercially available standards are limited, such as certain lipid families and carnitines, we leveraged the retention times of known standards and MS/MS fragmentation patterns of unknowns within the chemical family. As carbon chain lengths increase, predictable shifts in retention times and characteristic fragment ions allow for accurate identification across related compound class. Peak qualities were inspected for each annotated compound and peaks with high signal to noise ratios or insufficient MS1 scans for proper quantitation were removed.

Metabolomics data quality assurance and control

The analytical performance of the LC-MS systems and the quality of the metabolomics data were ensured through several strategies. Mixtures of synthetic reference standards, containing up to about 150 metabolites were analyzed before initiating analyses to ensure reproducibility of chromatographic retention times, quality of chromatographic peak shapes, and mass spectrometry (MS) sensitivity. These samples were monitored periodically during the analysis queue and were also used to confirm compound IDs. Internal standard signals were monitored in each sample to ensure proper injection and to monitor MS sensitivity. Pairs of pooled reference samples, created from the study samples, were inserted into the analysis queue at intervals of 20 study samples to determine reproducibility and data standardization. One pooled sample from each pair was used to calculate coefficients of variation (CVs) for every metabolite measured, and the second pooled sample is used to standardize data across the run using "nearest-neighbor" normalization.

Samples were stored at −80′C according to the "Standardized approaches for clinical sampling and endpoint ascertainment in tuberculous meningitis studies"[45] which should have prevented degradation. Metabolite concentrations over time are now indicated in Figure S6.

QUANTIFICATION AND STATISTICAL ANALYSIS

This study has two main objectives. The primary aim is to identify robust (universal) top-hit metabolites associated with mortality across two cohorts, while the secondary aim is to determine which metabolites have the strongest predictive value for mortality. However, untargeted metabolomics data face significant challenges due to their high dimensionality and the strong correlations between metabolites. High dimensional data lead to multiplicity (i.e., potential inflation of the type I error rate through multiple testing), and correlated metabolites tend to form clusters that may appear predictive but lack true biological associations, thereby contributing to false positive findings. Conventional statistical methods to control the False Discovery Rate (FDR), such as the Benjamini-Hochberg correction,[46] often overcompensate, increasing the likelihood of false negatives. Moreover, these methods do not account for sampling variability, resulting in inconsistent findings and reduced reproducibility.

A two-cohort validation strategy

To address these limitations, we employed a two-cohort validation strategy combined with a resampling ranking procedure[47] to ensure robust and reproducible findings (Figure S3).

(1) **Discovery Cohort (Indonesia):** The cohort was randomly split into two subsets of equal sizes:
(2) **Screening Set:** Used to identify potential top-hit metabolites associated with mortality through a resampling ranking procedure.
(3) **Within-Cohort Validation Set:** Tested the reproducibility of the identified metabolites within the same population, ensuring the stability of the signals under varying data subsets.
(4) **External Validation Cohort (Vietnam):** Served as an independent cohort to confirm the robustness of the findings, ensuring they were generalizable across different populations with distinct characteristics.

This dual-layered approach, inspired by the sure independence screening procedure,[48–50] validated signals both within the discovery cohort and in an independent external cohort. This strategy strengthened the reliability of the identified metabolites and demonstrated their applicability across diverse populations.

Metabolite data quality control and data preprocessing

In the analysis, we excluded metabolites with peak ion intensities below the detection limit in over 25% of TBM patients and control groups or with a coefficient of variation (CV) exceeding 30% in pooled samples. Values under the detection limit in the remaining patients, were substituted with half of the minimum measured value for that metabolite. If a metabolite was measured using multiple LC-MS methods, we selected the one with the lowest CV. All metabolites were then $\log_2$-transformed. For the ranking procedure, metabolite values were then normalized by their standard deviation. We excluded two patients with outlier metabolite values, using Wilkinson's algorithm for supervised outlier detection.[51]

Descriptive analysis

In descriptive analysis, we summarized and compared baseline patient characteristics and 60-day mortality between the two cohorts. We presented proportions for binary variables and median ($1^{st}/3^{rd}$ interquartile) for continuous variables. Categorical variables were tested with the chi-square test, continuous variables with the Wilcoxon rank-sum test, and mortality outcomes with the log rank test. We assessed and visualizing the correlation structure of all metabolites in each study population based on Spearman correlation matrix. Additionally, we compared metabolite abundance differences between TBM vs. non-infectious control using Wilcoxon rank-sum test, presenting them in a volcano plot. Furthermore, to visualize samples clustering in the CNS-disorder groups, we used principal component analysis on passed QC and normalized metabolites, and the first principal component was plotted against the second principal component. The 95% confidence ellipse was drawn for each group using multivariate *t*-distribution ellipse.

Survival analysis and resampling ranking procedure

In the primary analysis, we used a Cox regression model to assess the associations between metabolites with mortality, adjusting for age and HIV status. To identify the top-hits, we filtered the metabolites based on their correlation with two-month mortality ($p < 0.05$) and ranked the associations based on the logarithmic hazard ratio (log HR) using the screening subset ($n = 194$) from the Indonesian cohort. The resampling ranking procedure was applied to reduce the metabolite set to a manageable size while accounting for sampling variability. Specifically (Figure S5).

(1) **Sub-sampling:** A random 2/3 subset of the screening set was sampled 10,000 times.
(2) **Ranking:** Metabolites were ranked based on their hazard ratios (HR) in each resampling iteration.
(3) **Empirical Distribution:** The empirical ranking distribution was estimated for each of the 469 metabolites analyzed.

Top-ranking metabolites were selected based on a set of criteria to ensure that the metabolites demonstrated strong and consistent associations with mortality. To prevent bias, the selection was conducted blindly using metabolite identity codes. Metabolite

identities were unblinded only after the statistical analysis was completed. Specifically, selection criteria are described as follows, with thresholds (R1, R2) were determined relative to the total number of metabolites analyzed (K = 469).

(1) **Consistent Direction of Association:** 95% confidence intervals (CI) for HR consistently indicated an association ($p < 0.05$).
(2) **Rank Consistency (Lower Limit):** The 2.5% quantile of the rank distribution was below a threshold (R1 = 75).
(3) **Rank Consistency (Upper Limit):** The 97.5% quantile of the rank distribution was below a threshold (R2 = 225).

These top-hit metabolites were then validated in both the Indonesian cohort (n = 194) and the Vietnamese cohort (n = 679). To ensure we would not miss Vietnamese cohort-specific signals, we also conducted a sensitivity analysis in which the Vietnamese cohort was used for discovery and the complete Indonesian cohort for external validation. The combined list of signal metabolites from both analyses was considered the final associated metabolites. In addition, we conducted several secondary (exploratory) analyses as described below, in which we did not correct for multiple testing.

Pathway analysis

We performed a metabolite set enrichment analysis for predicting day 60 survival status, using metabolites with available HMDB ID. We used the MetaboAnalyst 6.0 platform.[42]

Clustering and association analysis of metabolites with clinical characteristics

We conducted hierarchical clustering analysis using Ward algorigthm implemented in hclust function in R to group metabolites and gain insights into the roles of the top-hit metabolites. We performed Spearman's correlation analysis to assess the associations between top-hit metabolites and patient characteristics, including age, Glasgow Coma Scale (GCS), CSF leukocyte counts, CSF/blood glucose ratio, CSF protein, CSF GeneXpert Ct value, and CSF cytokines. These cytokines (TNF-α, IFN-γ, IL-2, IL-4, IL-5, IL-6, IL-10, and IL-13) were measured using O-link in the Indonesian cohort and Luminex in the Vietnamese cohort and were analyzed separately.[13]

Comparison of metabolites abundance with other groups of meningitis patients

We assessed the disease-specific role of the metabolites by comparing their levels between survival and dead patients with various forms of CNS inflammatory diseases including bacterial (n = 50) and cryptococcal meningitis (n = 60), as well as non-infectious control (n = 54) as described in Ardiansyah et al.[13] We also represented metabolite distributions by meningitis type with violin and scatterplots, comparing differences between CNS-disorder's etiology using the Wilcoxon rank-sum test.

Variable selection analysis - Multivariate analysis

We first combined the two lists of validated top-hit metabolites, which were independently identified from the two cohorts (Indonesia and Vietnam). Then we performed variable selection on this combined list of top-hit metabolites on the combined data, identifying the most predictive metabolites alongside key clinical factors (GCS, GeneXpert Ct value, age, and HIV status). This analysis is based on a Gradient Boosting-Cox regression model using the stability selection approach proposed by Meinshausen and Bühlmann[23] and implemented in the R package ***stabs***.[35] This framework also controls for the per-family error rate (PFER) using the specified formula:

$$E(V) \leq \frac{q2}{(2\hat{\pi} - 1)p}$$

where,

(1) V is the number of variables wrongly selected by the procedure and $E(V)$ is the expected value of variables wrongly selected by the procedure
(2) q is the number of pre-anticipated selected variables
(3) $\hat{\pi}$ is the selected probability
(4) p is the number of variables.

In our analysis, we pre-specified $E(V) = 1\ (PFER), q = 6$, and $p = 16$. To address missing data in GeneXpert Ct value, we conducted a sensitivity analysis by excluding it to check result consistency.

Mediation analysis

To explore how TBM severity mediates the link between top-hit metabolites and TBM mortality, we assessed top-hit metabolite's trends in relation to TBM pre-treatment severity using Jonckheere-Terpstra tests, followed by a mediation analysis. This analysis utilized the causal diagram (Figure 2C), decomposing the total impact – TI of pre-treatment CSF top-hit metabolites on mortality into natural direct impact – NDI (β_X) and natural indirect impact – NII ($\alpha_X * \beta_M$) via TBM severity, based on the g-formula approach.[52] We calculated the proportion mediated (PM) coefficient as PM = NII/TI on the log hazard scale.

Mathematically, the key components of mediation analysis are described as follows:

CellPress
OPEN ACCESS

(1) *Total Impact – TI* (β'_X)*:* This is the overall impact of the variation of the independent variable *X* on the dependent variable *Y*, without considering any intermediary variables. It's typically represented by the coefficient β'_X in regression models.
(2) *Natural Direct Impact – NDI* (β_X)*:* This represents the impact of the variation in *X* on *Y* that is not mediated by the intermediate variable *M.* It s calculated after controlling (conditioning) for the impact of *M* on *Y.* It can be obtained by regressing *Y* on both *X* and *M* and then examining the coefficient of *X.*
(3) *Natural Indirect Impact – NII* ($\alpha_X * \beta_M$)*:* This is the impact of the variation of *X* on *Y* that operates through the intermediate variable *M.* It s calculated by multiplying the impact of X on M (a) by the impact of M on Y (b). The product of coefficients α_X and β_M is often referred to as the mediation impact or the indirect impact.
(4) *Proportion Mediated - PM: PM* $= \frac{\alpha_X * \beta_M}{\beta'_X}$.

Re-analysis of serum and CSF levels of top-hit metabolites for fatty acids and carnitines

Given the overrepresentation of fatty acids and their respective carnitine conjugates in the top-hit metabolites associated with mortality, we re-analyzed the abundances in paired serum-CSF samples of these top-hit metabolites from a previous cohort study. This cohort included both TBM patients ($n = 32$) and non-infectious controls ($n = 20$ (Serum); $n = 22$ (CSF)).[12] We used the same QC criteria as for untargeted metabolites, e.g., metabolites were excluded when the number of measurements below the detection limit exceeded 25% in either the TBM or control group. The abundances were $\log_2$ transformed. We computed and visualized the fold changes for the top-hit metabolites in both CSF and serum between TBM and non-infectious control patients with unadjusted *p*-values. The measurements reported for FA 6:0; OH represent the values from the isobaric FA 6:0; 3OH.

Furthermore, the annotation of carnitines was expanded in the current CSF dataset, increasing the total number of carnitines after QC from 16 to 33. These include monocarboxylic, dicarboxylic (DC), and hydroxy (OH) variants with an acyl length ranging from 2 to 32 or 2 tot 26 for DC, OH, and varying saturation (0–4 double bonds). The abundances of all annotated carnitines ($n = 33$) and fatty acids ($n = 48$) were $\log_2(x+1)$ transformed to visualize the relative abundances in the CSF across patient groups. The patient groups were compared (TBM, bacterial meningitis, and cryptococcal meningitis patients vs. non-infectious control patients) based on Wilcoxon statistical test, corrected for FDR ($n = 243$, accounting for the between patient group comparisons). Fold changes were computed and corrected for FDR ($n = 81$, total number of carnitines and fatty acids) within a pairwise comparison, including the comparison non-survivors versus survivors within TBM for day 60. All fold change values are shown. Furthermore, the Spearman correlation coefficient between these metabolites and CSF protein levels as well as bacterial load was computed.

Med, Volume *6*

Supplemental information

Pre-treatment untargeted cerebrospinal fluid metabolomic profiling in tuberculous meningitis uncovers pathways associated with mortality

Thanh Hoang Nhat Le, Kirsten C.J. van Abeelen, Edwin Ardiansyah, Julian Avila-Pacheco, Sofiati Dian, Gesa Carstens, Lara Schramke, Hoang Thanh Hai, Tran Binh Minh Nguyen, Thai Minh Triet, Amy Deik, Jesse Krejci, Jeff Pruyne, Lucas Dailey, Bachti Alisjahbana, Mihai G. Netea, Riwanti Estiasari, Trinh Thi Bich Tram, Joseph Donovan, Dorothee Heemskerk, Thi Hong Chau Tran, Nguyen Duc Bang, Ahmad Rizal Ganiem, Raph L. Hamers, Rovina Ruslami, Darma Imran, Kartika Maharani, Vinod Kumar, Reinout van Crevel, Guy Thwaites, Clary B. Clish, Nguyen Thuy Thuong Thuong, and Arjan van Laarhoven

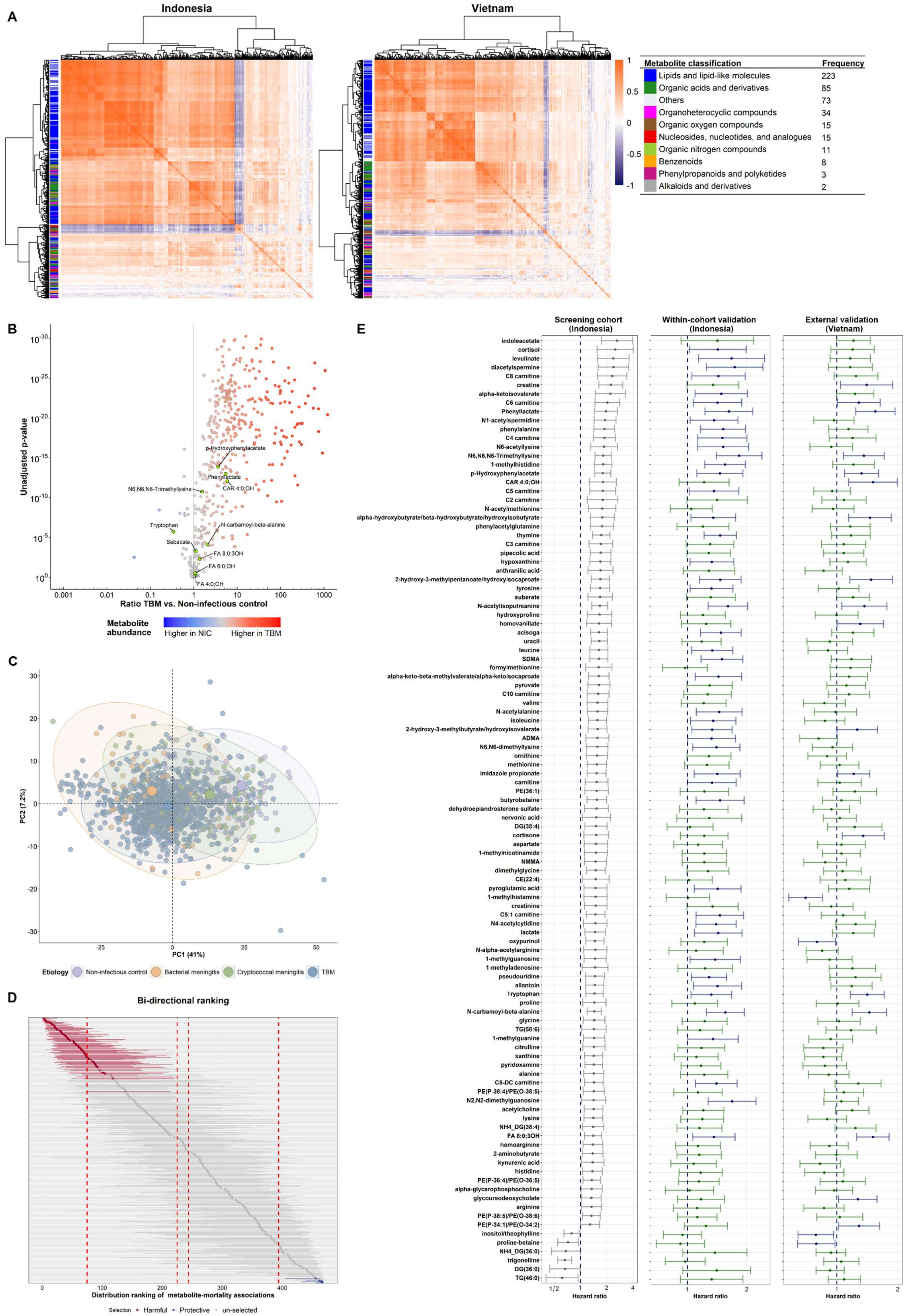
A
Indonesia
Vietnam
1
0.5
0
-0.5
-1
Metabolite classification
Frequency
Lipids and lipid-like molecules 223
Organic acids and derivatives 85
Others 73
Organoheterocyclic compounds 34
Organic oxygen compounds 15
Nucleosides, nucleotides, and analogues 15
Organic nitrogen compounds 11
Benzenoids 8
Phenylpropanoids and polyketides 3
Alkaloids and derivatives 2
B
Unadjusted p-value
Ratio TBM vs. Non-infectious control
p-Hydroxyphenylacetate
Phenyllactate
CAR 4:0;OH
N6,N6,N6-Trimethyllysine
Tryptophan
N-carbamoyl-beta-alanine
Sebacate
FA 8:0;3OH
FA 6:0;OH
FA 4:0;OH
Metabolite abundance
Higher in NIC
Higher in TBM
C
PC1 (41%)
PC2 (7.2%)
Etiology
Non-infectious control
Bacterial meningitis
Cryptococcal meningitis
TBM
D
Bi-directional ranking
Distribution ranking of metabolite-mortality associations
Selection
Harmful
Protective
un-selected
E
Screening cohort (Indonesia)
Within-cohort validation (Indonesia)
External validation (Vietnam)
Hazard ratio
indoleacetate
cortisol
levulinate
diacetylspermine
C8 carnitine
creatine
alpha-ketoisovalerate
C6 carnitine
Phenyllactate
N1-acetylspermidine
phenylalanine
C4 carnitine
N6-acetyllysine
N6,N6,N6-Trimethyllysine
1-methylhistidine
p-Hydroxyphenylacetate
CAR 4:0;OH
C5 carnitine
C2 carnitine
N-acetylmethionine
alpha-hydroxybutyrate/beta-hydroxybutyrate/hydroxyisobutyrate
phenylacetylglutamine
thymine
C3 carnitine
pipecolic acid
hypoxanthine
anthranilic acid
2-hydroxy-3-methylpentanoate/hydroxyisocaproate
tyrosine
suberate
N-acetylisoputreanine
hydroxyproline
homovanillate
acisoga
uracil
leucine
SDMA
formylmethionine
alpha-keto-beta-methylvalerate/alpha-ketoisocaproate
pyruvate
C10 carnitine
valine
N-acetylalanine
isoleucine
2-hydroxy-3-methylbutyrate/hydroxyisovalerate
ADMA
N6,N6-dimethyllysine
ornithine
methionine
imidazole propionate
carnitine
PE(36:1)
butyrobetaine
dehydroepiandrosterone sulfate
nervonic acid
DG(38:4)
cortisone
aspartate
1-methylnicotinamide
NMMA
dimethylglycine
CE(22:4)
pyroglutamic acid
1-methylhistamine
creatinine
C5:1 carnitine
N4-acetylcytidine
lactate
oxypurinol
N-alpha-acetylarginine
1-methylguanosine
1-methyladenosine
pseudouridine
allantoin
Tryptophan
proline
N-carbamoyl-beta-alanine
glycine
TG(58:6)
1-methylguanine
citrulline
xanthine
pyridoxamine
alanine
C5-DC carnitine
PE(P-38:4)/PE(O-38:5)
N2,N2-dimethylguanosine
acetylcholine
lysine
NH4_DG(38:4)
FA 8:0;3OH
homoarginine
2-aminobutyrate
kynurenic acid
histidine
PE(P-36:4)/PE(O-36:5)
alpha-glycerophosphocholine
glycoursodeoxycholate
arginine
PE(P-38:5)/PE(O-38:6)
PE(P-34:1)/PE(O-34:2)
inositol/theophylline
proline-betaine
NH4_DG(36:0)
trigonelline
DG(36:0)
TG(46:0)

Figure S1: The association of metabolites with TBM mortality, related to Figure 1

(A) Heatmap of Spearman correlations of 469 metabolites. Each panel for each cohort. Hierarchical clustering analysis based on their Spearman correlation structure. The table summarizes the count of metabolites within each metabolite classification (HMDB: the Human Metabolome Database, Nucleic Acids Res. 2007 Jan; 35:D521-6). See also Table S8.

(B) Volcano plot showing the fold change of 469 metabolites between TBM and non-infectious control groups, with unadjusted p-values from the Wilcoxon rank sum test. Among these, 400 metabolites are significantly higher in TBM ($p < 0.05$), while 23 are significantly higher in NIC. Green dots represent the top-hit metabolites.

(C) Scatter plots of projection of patients' metabolites onto the two main principal components. The 95% confidence ellipse was plotted for diagnostic group using multivariate t-distribution ellipse. PC = principal component.

(D) Figure shows the ranking plot of 469 metabolites in the screening set, visualizing the distribution of the magnitude of metabolite-mortality associations. Each dot and its error bars represent the median rank and the 2.5% and 97.5% quantiles of the rank for each metabolite. Based on the three criteria, 107 metabolites were selected as the top-ranking metabolites in the screening set. The red dots (and error bars) correspond to harmful metabolites (i.e., HR estimate > 1), while the dark blue dots (and error bars) correspond to protective metabolites (i.e., HR estimate < 1). Metabolites not selected by the three criteria are shown in grey.

(E) The Panel 1, Panel 2 and Panel 3 corresponds to the forest plots of 107 selected metabolites as the top-ranking metabolites in the screening set (Indonesia), within cohort validation set (Indonesia) and the external validation set (Vietnam). In the three forest plots, each dot shows hazard ratio estimates (with confidence interval) of an increase of one standard deviation of abundance of the respective metabolites. Blue indicates $P<0.05$ and green $P\geq0.05$.

S2.1. Fatty Acids

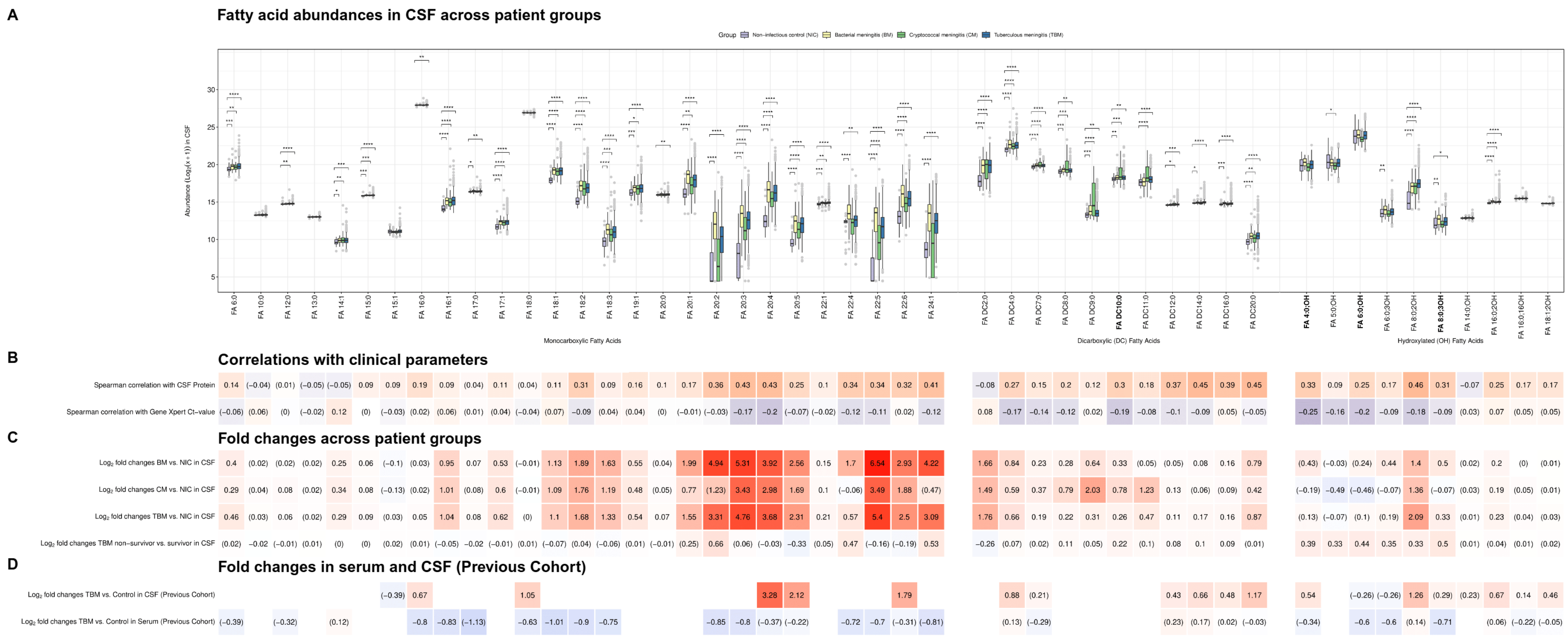

S2.2. Carnitine

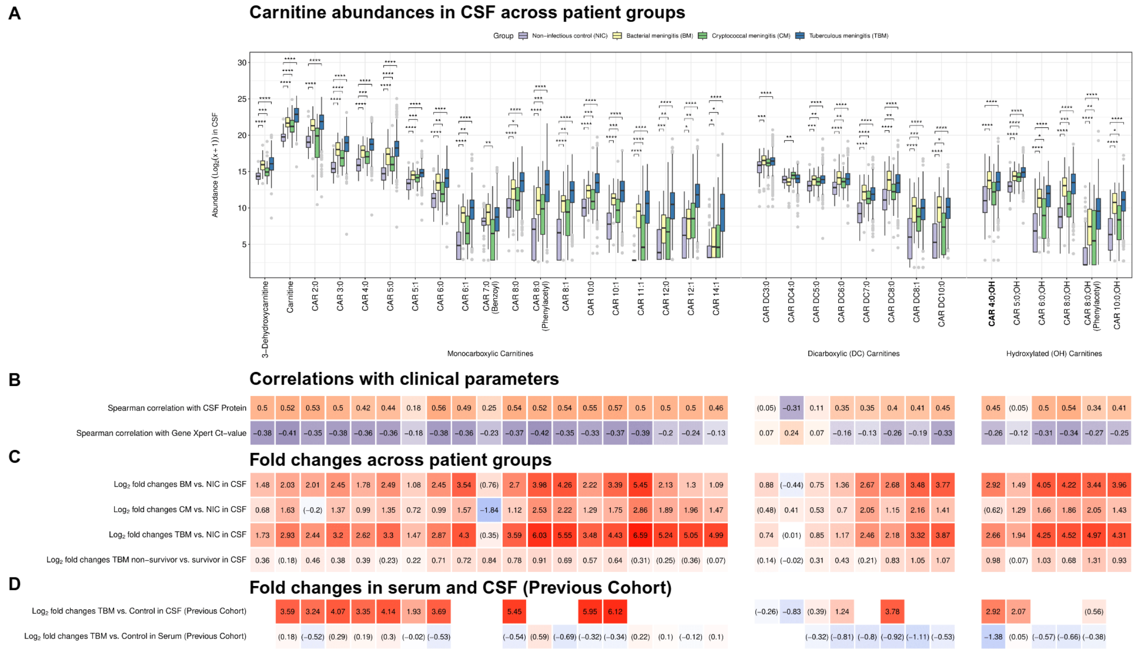

Figure S2: Relative abundance circulating fatty acids and carnitines in CSF across patient groups, related to Figure 3

(2.1) Abundances of available fatty acids in CSF and serum across patient groups and Spearman correlations with clinical parameters for TBM. Fatty acids are ordered by their length, number of double bonds, and class: mono-carboxylic, dicarboxylic (DC), and having a hydroxy (OH) group. Metabolites identified in the primary analysis are highlighted in bold. Panel A: Distributions of fatty acids where asterisks denote the significance levels following Wilcoxon statistical test (**** < $1x10^{-4}$, *** [$1x10^{-4}$, 0.001], ** [0.001, 0.01], * [0,01, 0.05]), where patient groups are compared to the non-infectious control (NIC) group. Panel B: Spearman correlations with CSF protein and bacterial load (GeneXpert Ct-value) for the TBM group, where correlations with unadjusted p-value > 0.05 are denoted by in parentheses. Panel C: Log_2 fold changes for TBM, bacterial meningitis (BM), and cryptococcal meningitis (CM) patients vs. NIC as well as non-survivors vs. survivors within TBM. Fold changes with adjusted p-value > 0.05 are enclosed in parentheses. Panel D: Log_2 fold changes for TBM patients vs. non-infectious control in CSF and serum, re-analyzed from the previous cohort (van Laarhoven et al., 2018). Fold changes with unadjusted p-value > 0.05 are enclosed in parentheses. Fold changes for fatty acids not measured in this data or not passing QC are left blank.

(2.2) Abundances of available carnitines in CSF and serum across patient groups and Spearman correlations with clinical parameters for TBM. Carnitines are ordered by their length, number of double bonds, and class: mono-carboxylic, dicarboxylic (DC), and having a hydroxy (OH) group. The analysis equals that in Figure S2.1.

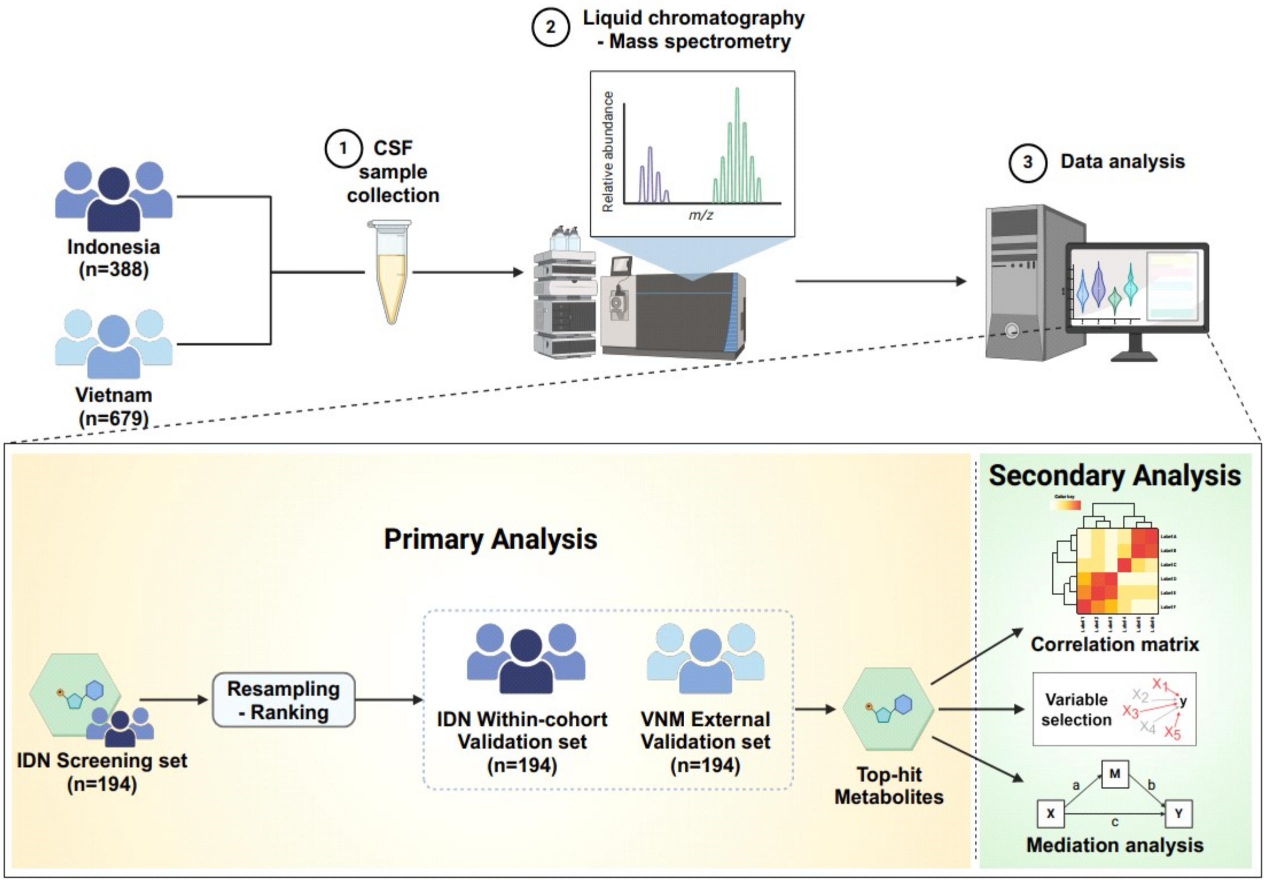

Figure S3. Analysis workflow diagram, related to STAR Methods

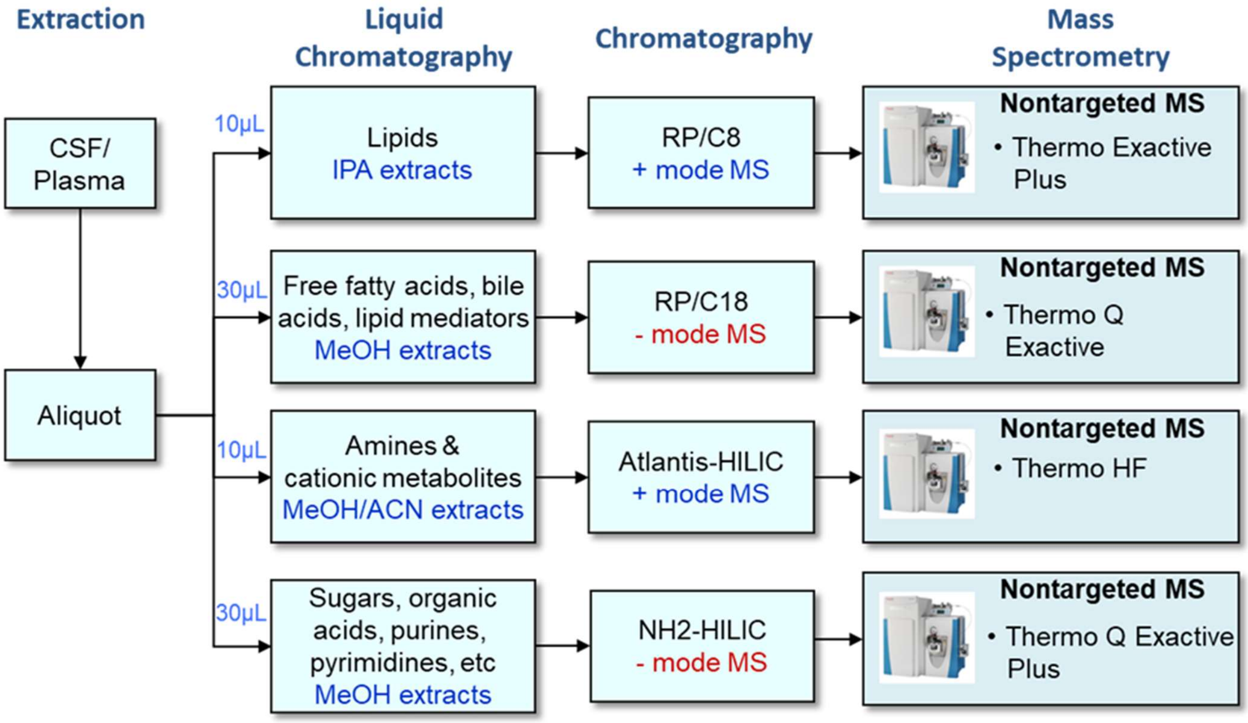

Figure S4. LC-MS-based analytical platform, related to STAR Methods

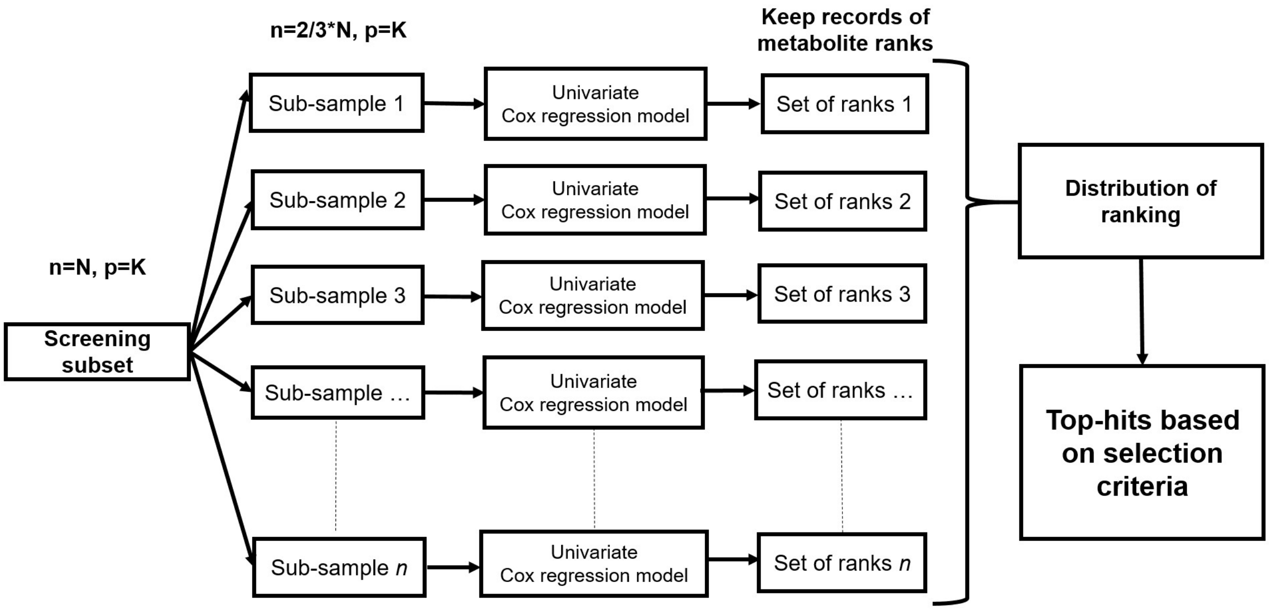

Figure S5. Sub-sampling ranking procedure, related to STAR Methods

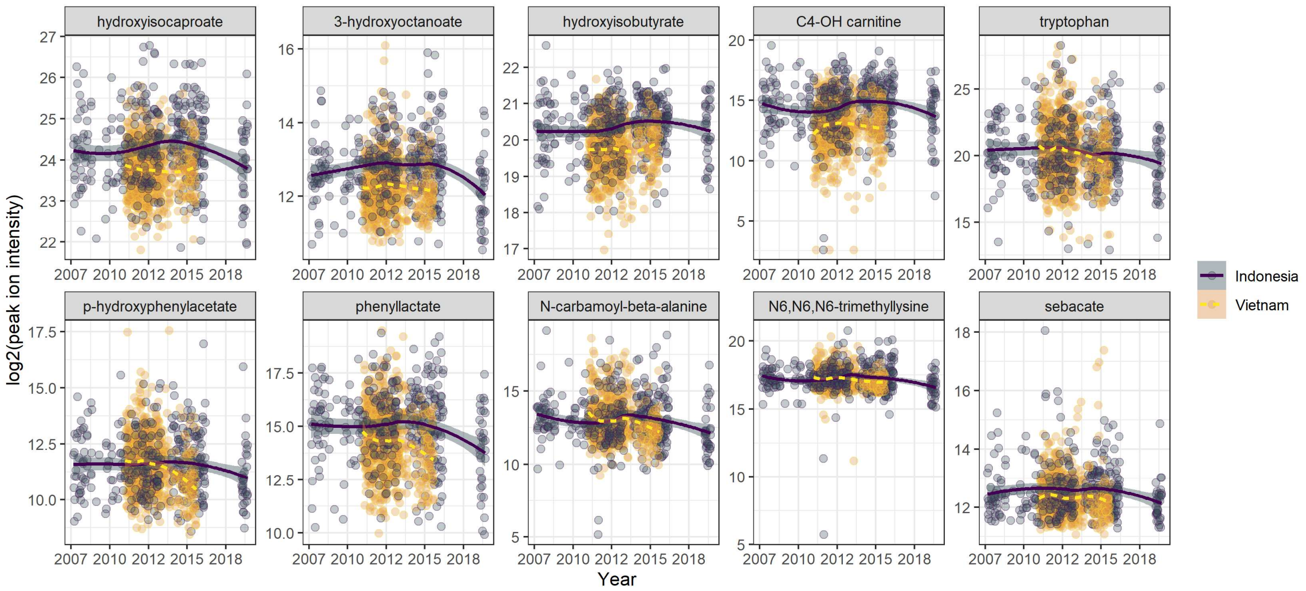

Figure S6. Top-hit metabolite's stability over time, related to STAR Methods

Table S1: Clinical characteristics association with TBM mortality-univariate analysis, related to Table 1

Characteristic	Indonesia Cohort				Vietnam Cohort			
	N	HR[1]	95% CI[1]	p-value	N	HR[1]	95% CI[1]	p-value
Sex	387				679			
Female		—	—			—	—	
Male		1.48	1.02, 2.14	0.038		1.09	0.72, 1.67	0.68
Age	387	1.03	1.01, 1.04	<0.001	679	1.02	1.00, 1.03	0.017
Diagnostic category	387				679			
Definite TBM		—	—			—	—	
Probable TBM		1.01	0.71, 1.44	0.94		1.7	1.15, 2.50	0.008
HIV status	387				679			
HIV-negative		—	—			—	—	
HIV-positive		2.32	1.44, 3.75	<0.001		2.39	1.62, 3.52	<0.001
Modified MRC disease-severity grade	368				679			
I		—	—			—	—	
II		1.84	0.74, 4.54	0.19		2.29	1.31, 4.02	0.004
III		5.25	2.05, 13.5	<0.001		6.53	3.72, 11.5	<0.001
Glasgow Coma Scale	387	0.79	0.73, 0.84	<0.001	679	0.81	0.77, 0.86	<0.001
**CSF Parameters**								
Total leukocytes — per 2-fold increase	387	0.97	0.90, 1.05	0.47	678	0.82	0.75, 0.90	<0.001
Polymorphonuclear leukocytes counts — per 2-fold increase	387	1.07	1.00, 1.15	0.048	653	0.96	0.90, 1.03	0.25
Mononuclear cells — per 2-fold increase	387	0.91	0.84, 0.99	0.024	653	0.82	0.74, 0.92	<0.001
Protein level — per 2-fold increase	387	1.12	1.01, 1.25	0.04	671	0.97	0.80, 1.17	0.71
CSF to blood glucose ratio	387	0.23	0.07, 0.77	0.018	671	0.81	0.30, 2.21	0.69
Mean GeneXpert Ct value	126	0.99	0.95, 1.02	0.45	654	1	0.97, 1.03	0.83

[1]HR = Hazard Ratio, CI = Confidence Interval

Table S2: Patient's characteristics of TBM in compared with other meningitis, related to Table 1

Characteristics	n[1]	Bacterial meningitis, N = 50[1,2]	n[1]	Cryptococcal meningitis, N = 62[1,2]	n[1]	Non-infectious control, N = 54[1,2]	n[1]	Tuberculous meningitis, N = 1,067[1,2]	p-value[3]
Female sex — no. (%)	20	8 (40%)	35	7 (20%)	54	24 (44%)	1067	369 (65%)	0.12
Age — median (1st and 3rd interquartile) (years)	20	46 (34, 57)	56	33 (27, 37)	54	35 (25, 44)	1067	34 (27, 44)	0.012
HIV-positive — no. (%)	50	0 (0%)	61	60 (98%)	54	11 (20%)	1067	284 (27%)	<0.001
**CSF parameter**									
Total leukocytes count — cells/µL	35	1,900 (739, 5,460)	35	103 (28, 201)	54	2 (1, 3)	1066	150 (49, 336)	<0.001
Polymorphonuclear leukocytes counts—cells/µL	34	1,527 (538, 4,986)	35	17 (6, 97)	46	1 (0, 1)	1041	22 (3, 100)	<0.001
Mononuclear cells — cells/µL	34	307 (134, 646)	35	36 (7, 89)	46	2 (1, 3)	1041	98 (38, 206)	<0.001
Protein level, g/L	35	190 (110, 380)	35	76 (58, 167)	54	40 (26, 59)	1059	145 (90, 240)	<0.001
CSF to blood glucose ratio	32	0.30 (0.10, 0.47)	35	0.30 (0.20, 0.40)	54	0.60 (0.56, 0.70)	1059	0.28 (0.17, 0.40)	<0.001
Median Glasgow coma scale (1st and 3rd interquartile)	20	13 (9, 14)	35	15 (13, 15)	54	15 (12, 15)	1067	14 (12, 15)	0.01

[1]Number of non-missing observations; median (1st and 3rd interquartile) for continuous variables and frequency (%) for categorical variables.
[2]Patients lost to follow-up included 7 with BM, 12 with CM, 21 in the Non-infectious control group, and 12 with TBM. Additionally, 30 BM patients and 27 CM patients did not have follow-up.
[3]Pearson's chi-squared test for category variables; Wilcoxon rank sum test for continuous variables.

Table S3: Association of top-hit metabolites with 6-month mortality in both cohorts, related to Table 2

Metabolite	Indonesia Cohort				Vietnam Cohort			
	N	HR[1]	95% CI[1]	p-value	N	HR[1]	95% CI[1]	p-value
FA 6:0;OH	387	1.46	1.26, 1.69	<0.001	679	1.48	1.21, 1.82	<0.001
FA 8:0;3OH	385	1.35	1.17, 1.57	<0.001	678	1.63	1.41, 1.90	<0.001
FA 4:0;OH	387	1.44	1.21, 1.72	<0.001	679	1.37	1.12, 1.66	0.002
CAR 4:0;OH	386	1.37	1.12, 1.67	0.003	679	1.46	1.17, 1.82	<0.001
Tryptophan	386	1.34	1.16, 1.55	<0.001	679	1.56	1.34, 1.82	<0.001
p-Hydroxyphenylacetate	385	1.55	1.33, 1.81	<0.001	678	1.44	1.24, 1.68	<0.001
Phenyllactate	385	1.54	1.28, 1.85	<0.001	678	1.71	1.43, 2.06	<0.001
N-carbamoyl-beta-alanine	386	1.43	1.25, 1.65	<0.001	679	1.63	1.40, 1.89	<0.001
N6,N6,N6-Trimethyllysine	386	1.77	1.51, 2.08	<0.001	679	1.44	1.19, 1.75	<0.001
Sebacate	387	1.23	1.09, 1.39	<0.001	679	1.33	1.16, 1.52	<0.001

[1]HR = Hazard Ratio, CI = Confidence Interval

Table S4: Pathway enrichment analysis results for day 60 mortality, related to Figure 1 and Table 2

Pathway	Total Cmpd	Hits	Statistic Q	Expected Q	Raw p	FDR
Pyrimidine metabolism	39	6	30,521	0.0943	7.85e-13	4.79e-10
Tryptophan metabolism	41	5	2,013	0.0943	4.18e-08	1.27e-06
Glycerolipid metabolism	16	2	23,571	0.0943	1.03e-05	0.0002
Sphingolipid metabolism	32	4	25,504	0.0943	1.08e-05	0.0002
Tyrosine metabolism	42	5	18,077	0.0943	3.16e-08	0.0039
Galactose metabolism	27	4	12,544	0.0943	0.0062	0.0628
Ascorbate and aldarate metabolism	9	2	17,397	0.0943	2.13e-06	0.1624
Inositol phosphate metabolism	30	2	17,397	0.0943	2.13e-06	0.1624
Pantothenate and CoA biosynthesis	20	6	11,218	0.0943	0.0345	0.2304
Pyruvate metabolism	23	2	1,313	0.0943	0.0378	0.2304
Pentose and glucuronate interconversions	19	1	18,944	0.0943	0.0675	0.3742
Glycolysis / Gluconeogenesis	26	1	1,823	0.0943	0.1010	0.5132
Neomycin, kanamycin and gentamicin biosynthesis	2	1	17,233	0.0943	0.1774	0.8326
beta-Alanine metabolism	21	4	10,942	0.0943	0.1995	0.8694
Citrate cycle (TCA cycle)	20	5	0.7842	0.0943	0.2581	0.0001
Phenylalanine metabolism	8	2	14,043	0.0943	0.6173	0.0002
Phenylalanine, tyrosine and tryptophan biosynthesis	4	2	14,043	0.0943	0.6173	0.0002
Valine, leucine and isoleucine degradation	39	6	11,003	0.0943	0.0001	0.0004
Purine metabolism	70	9	0.5145	0.0943	0.0001	0.0004
Steroid hormone biosynthesis	87	4	0.8380	0.0943	0.0002	0.0006
Valine, leucine and isoleucine biosynthesis	8	6	10,457	0.0943	0.0002	0.0007
Lipoic acid metabolism	28	2	0.7638	0.0943	0.0013	0.0036
Pentose phosphate pathway	23	1	0.9059	0.0943	0.0019	0.0048
Lysine degradation	30	3	0.7645	0.0943	0.0019	0.0048
Starch and sucrose metabolism	18	3	0.6969	0.0943	0.0025	0.0060
Caffeine metabolism	10	1	0.8553	0.0943	0.0026	0.0060
Primary bile acid biosynthesis	46	7	0.6702	0.0943	0.0029	0.0065
Ubiquinone and other terpenoid-quinone biosynthesis	18	1	0.8082	0.0943	0.0034	0.0073
Glycerophospholipid metabolism	36	5	0.5343	0.0943	0.0042	0.0089
Amino sugar and nucleotide sugar metabolism	42	1	0.7393	0.0943	0.0050	0.0103
Glycine, serine and threonine metabolism	33	10	0.4562	0.0943	0.0072	0.0141
Taurine and hypotaurine metabolism	8	2	0.5028	0.0943	0.0171	0.0325
Histidine metabolism	16	6	0.2961	0.0943	0.0204	0.0378
Cysteine and methionine metabolism	33	5	0.3306	0.0943	0.0255	0.0457
Glyoxylate and dicarboxylate metabolism	31	8	0.2977	0.0943	0.0279	0.0487
Butanoate metabolism	15	3	0.3390	0.0943	0.0356	0.0603
Thiamine metabolism	7	1	0.4048	0.0943	0.0382	0.0629

Table S5: Metabolites associated with mortality, related to Figure 1 and Table 2

Metabolite	Description	Biological effect and related metabolic pathways
FA 6:0;OH<br>(hydroxy-isocaproate)<br>$C_6H_{12}O_3$	Hydroxy-carboxylic acid	• Beta-oxidation intermediate
FA 8:0;3OH<br>(3-hydroxyoctanoate)<br>$C_8H_{16}O_3$	Hydroxy-carboxylic acid	• Beta-oxidation intermediate<br>• Ligand for GPR109B (HCA3), a GPCR expressed in adipocytes and immune cells (Ahmed et al., 2009)<br>• HCA3 ligands suppress cytokine expression (Mandrika et al., 2018)
FA 4:0;OH<br>(alpha-hydroxybutyrate or beta-hydroxybutyrate)<br>$C_4H_8O_3$	Hydroxy-carboxylic acid	• Beta-oxidation intermediate<br>• Ketone body
CAR 4:0;OH<br>(3-hydroxybutyrylcarnitine)<br>$C_{11}H_{21}NO_5$	Conjugate of FA 4:0;OH and carnitine	• Beta-oxidation ketone body metabolite<br>• Elevated in plasma in response to starvation (Steinhauser et al., 2018)
Tryptophan<br>$C_{11}H_{12}N_2O_2$	Amino acid	• Precursor of serotonin and kynurenine
p-Hydroxyphenylacetate<br>$C_8H_8O_3$		• Downstream metabolites of the transamination product of phenylalanine, phenylpyruvate (Antoshechkin et al., 1991; van Spronsen et al., 2021)
Phenyllactate<br>$C_9H_{10}O_3$	Phenylalanine catabolite; produced from phenylpyruvate via the action of lactate dehydrogenase	• Antimicrobial compound synthesized by lactic acid bacteria<br>• Decreases ROS production in mitochondria and neutrophils (Beloborodova et al., 2012)<br>• Downstream metabolites of the transamination product of phenylalanine, phenylpyruvate (Antoshechkin et al., 1991; van Spronsen et al., 2021)
N-carbamoyl-beta-alanine<br>(3-Ureidopropionic acid)<br>$C_4H_8N_2O_3$	An intermediate in the metabolism of uracil	• Inhibits respiratory chain complex V and may function as an endogenous neurotoxin in the context of 3-ureidopropionase deficiency (Kölker et al., 2001)<br>• Higher plasma levels linked to heart failure risk (Yazdani et al., 2024) and frailty (Marron et al., 2019)
Sebacic acid<br>(sebacate)<br>$C_{10}H_{18}O_4$	Dicarboxylic acid	• Elevated in urine of patients with medium-chain acyl-CoA dehydrogenase deficiency (Gregersen et al., 1983)
N6,N6,N6-trimethyllysine<br>$C_9H_{20}N_2O_2$	Methylated derivative of lysine	• Precursor for carnitine biosynthesis

Table S6: Distribution of log2-transformed peak ion abundances for the metabolites associated to outcome, stratified by TBM severity (MRC grade), related to Figure 2, and stratified by HIV groups, related to Figure 4

Metabolites	TBM severity (MRC grade)				HIV Groups		
	**Grade I**, N = 287[1]	**Grade II**, N = 582[1]	**Grade III**, N = 179[1]	**p-value**[2]	**HIV-negative**, N = 783[1]	**HIV-positive**, N = 284[1]	**p-value**[3]
FA 6:0;OH	28.88 (28.41, 29.39)	29.34 (28.73, 30.01)	29.55 (29.11, 30.14)	<0.0001	29.33 (28.76, 30.02)	28.99 (28.46, 29.52)	<0.001
FA 8:0;3OH	14.62 (14.06, 15.11)	15.10 (14.45, 15.74)	15.14 (14.53, 15.67)	<0.0001	15.02 (14.37, 15.69)	14.76 (14.32, 15.38)	0.004
FA 4:0;OH	25.11 (24.52, 25.61)	25.76 (25.11, 26.39)	25.98 (25.35, 26.47)	<0.0001	25.72 (25.01, 26.40)	25.35 (24.72, 25.90)	<0.001
CAR 4:0;OH	5.29 (4.75, 5.79)	5.85 (5.37, 6.41)	5.93 (5.36, 6.48)	<0.0001	5.80 (5.27, 6.41)	5.53 (4.96, 6.01)	<0.001
Tryptophan	8.58 (7.96, 9.41)	8.73 (8.17, 9.54)	9.05 (8.38, 9.49)	0.0003	8.69 (8.08, 9.42)	8.92 (8.31, 9.67)	0.001
p-hydroxyphenylacetate	8.34 (7.84, 8.98)	8.53 (7.96, 9.30)	8.97 (8.30, 9.68)	<0.0001	8.50 (7.93, 9.24)	8.69 (8.07, 9.28)	0.077
Phenyllactate	7.88 (7.24, 8.42)	8.32 (7.68, 8.89)	8.66 (8.08, 9.17)	<0.0001	8.25 (7.60, 8.84)	8.33 (7.58, 8.86)	0.6
N-carbamoyl-beta-alanine	8.51 (8.13, 8.94)	8.58 (8.07, 9.16)	8.84 (8.25, 9.71)	0.001	8.50 (8.05, 9.07)	8.83 (8.36, 9.50)	<0.001
N6,N6,N6-Trimethyllysine	20.29 (19.91, 20.65)	20.45 (19.95, 20.98)	20.67 (20.09, 21.29)	<0.0001	20.42 (19.95, 20.96)	20.38 (19.92, 20.86)	0.3
Sebacate	31.26 (30.91, 31.63)	31.35 (30.96, 31.85)	31.44 (31.07, 31.96)	0.0001	31.32 (30.95, 31.83)	31.40 (31.05, 31.79)	0.4

[1]*Median (1st, 3rd interquartile)*
[2]*p-value derived from trend test (Jonckheere-Terpstra test)*
[3]*p-value derived from Wilcoxon rank sum test*

Table S7: Causal mediation analysis of the impact of the pre-treatment metabolites to mortality by TBM severity, related to Figure 2

Metabolite	Total impact			Natural Direct Impact			Natural Indirect Impact			Proportion Mediated		
	HR[1]	95% CI[1]	p-value	HR[1]	95% CI[1]	p-value	HR[1]	95% CI[1]	p-value	HR[1]	95% CI[1]	p-value
FA 6:0;OH	1.67	1.42; 1.95	<0.001	1.67	1.42; 1.95	<0.001	1	1.00; 1.00	1	0	0.00; 0.00	1
FA 8:0;3OH	1.59	1.39; 1.84	<0.001	1.57	1.37; 1.82	<0.001	1.01	1.00; 1.04	0.061	0.04	0.00; 0.1	0.064
FA 4:0;OH	1.59	1.36; 1.89	<0.001	1.59	1.36; 1.89	<0.001	1	1.00; 1.00	1	0	0.00; 0.00	1
CAR 4:0;OH	1.77	1.46; 2.2	<0.001	1.62	1.35; 2.01	<0.001	1.09	1.04; 1.17	<0.001	0.19	0.09; 0.33	<0.001
Tryptophan	1.48	1.3; 1.69	<0.001	1.37	1.22; 1.57	<0.001	1.08	1.03; 1.12	<0.001	0.21	0.09; 0.33	0.001
p-Hydroxyphenylacetate	1.41	1.21; 1.65	<0.001	1.31	1.14; 1.53	<0.001	1.07	1.03; 1.13	<0.001	0.23	0.10; 0.39	<0.001
Phenyllactate	1.61	1.34; 1.94	<0.001	1.54	1.29; 1.87	<0.001	1.04	1.01; 1.09	<0.001	0.11	0.03; 0.21	<0.001
N-carbamoyl-beta-alanine	1.6	1.36; 1.86	<0.001	1.48	1.28; 1.72	<0.001	1.08	1.03; 1.12	<0.001	0.19	0.07; 0.29	0.001
N6,N6,N6-Trimethyllysine	1.56	1.32; 1.83	<0.001	1.55	1.32; 1.83	<0.001	1	1.00; 1.01	0.444	0.01	-0.01; 0.03	0.454
Sebacate	1.26	1.14; 1.45	<0.001	1.26	1.14; 1.45	<0.001	1	1.00; 1.01	0.589	-0.01	-0.02; 0.05	0.609

[1]HR = Hazard Ratio, CI = Confidence Interval

Supplemental references

1. van Laarhoven, A. et al. Cerebral tryptophan metabolism and outcome of tuberculous meningitis: an observational cohort study. Lancet Infect. Dis. 18, 526–535 (2018).
2. Ahmed, K., Tunaru, S., Langhans, C.-D., Hanson, J., Michalski, C. W., Kölker, S., Jones, P. M., Okun, J. G., & Offermanns, S. (2009). Deorphanization of GPR109B as a receptor for the beta-oxidation intermediate 3-OH-octanoic acid and its role in the regulation of lipolysis. The Journal of Biological Chemistry, 284(33), 21928–21933. https://doi.org/10.1074/jbc.M109.019455
3. Antoshechkin, A. G., Chentsova, T. V, VYu, T., Naritsin, D. B., & Railian, G. P. (1991). Content of phenylalanine, tyrosine and their metabolites in CSF in phenylketonuria. Journal of Inherited Metabolic Disease, 14(5), 749–754. https://doi.org/10.1007/BF01799945
4. Beloborodova, N., Bairamov, I., Olenin, A., Shubina, V., Teplova, V., & Fedotcheva, N. (2012). Effect of phenolic acids of microbial origin on production of reactive oxygen species in mitochondria and neutrophils. Journal of Biomedical Science, 19(1), 89. https://doi.org/10.1186/1423-0127-19-89
5. Gregersen, N., Kølvraa, S., Rasmussen, K., Mortensen, P. B., Divry, P., David, M., & Hobolth, N. (1983). General (medium-chain) acyl-CoA dehydrogenase deficiency (non-ketotic dicarboxylic aciduria): quantitative urinary excretion pattern of 23 biologically significant organic acids in three cases. Clinica Chimica Acta; International Journal of Clinical Chemistry, 132(2), 181–191. https://doi.org/10.1016/0009-8981(83)90246-2
6. Kölker, S., Okun, J. G., Hörster, F., Assmann, B., Ahlemeyer, B., Kohlmüller, D., Exner-Camps, S., Mayatepek, E., Krieglstein, J., & Hoffmann, G. F. (2001). 3-Ureidopropionate contributes to the neuropathology of 3-ureidopropionase deficiency and severe propionic aciduria: A Hypothesis. Journal of Neuroscience Research, 66(4), 666–673. https://doi.org/10.1002/jnr.10012
7. Mandrika, I., Tilgase, A., Petrovska, R., & Klovins, J. (2018). Hydroxycarboxylic Acid Receptor Ligands Modulate Proinflammatory Cytokine Expression in Human Macrophages and Adipocytes without Affecting Adipose Differentiation. Biological & Pharmaceutical Bulletin, 41(10), 1574–1580. https://doi.org/10.1248/bpb.b18-00301
8. Marron, M. M., Harris, T. B., Boudreau, R. M., Clish, C. B., Moore, S. C., Murphy, R. A., Murthy, V. L., Sanders, J. L., Shah, R. V, Tseng, G. C., Wendell, S. G., Zmuda, J. M., & Newman, A. B. (2019). Metabolites Associated with Vigor to Frailty Among Community-Dwelling Older Black Men. Metabolites, 9(5). https://doi.org/10.3390/metabo9050083
9. Steinhauser, M. L., Olenchock, B. A., O'Keefe, J., Lun, M., Pierce, K. A., Lee, H., Pantano, L., Klibanski, A., Shulman, G. I., Clish, C. B., & Fazeli, P. K. (2018). The circulating metabolome of human starvation. JCI Insight, 3(16). https://doi.org/10.1172/jci.insight.121434
10. van Spronsen, F. J., Blau, N., Harding, C., Burlina, A., Longo, N., & Bosch, A. M. (2021). Phenylketonuria. Nature Reviews. Disease Primers, 7(1), 36. https://doi.org/10.1038/s41572-021-00267-0
11. Yazdani, A., Mendez-Giraldez, R., Yazdani, A., Wang, R.-S., Schaid, D. J., Kong, S. W., Hadi, M. R., Samiei, A., Samiei, E., Wittenbecher, C., Lasky-Su, J., Clish, C. B., Muehlschlegel, J. D., Marotta, F., Loscalzo, J., Mora, S., Chasman, D. I., Larson, M. G., & Elsea, S. H. (2024). Broadcasters, receivers, functional groups of metabolites, and the link to heart failure by revealing metabolomic network connectivity. Metabolomics : Official Journal of the Metabolomic Society, 20(4), 71. https://doi.org/10.1007/s11306-024-02141-y
